# Biomaterials Based on Jellyfish Collagen from the Black Sea and Other Marine-Derived Biocomposites with Medical Applications

**DOI:** 10.3390/md24100338

**Published:** 2026-09-27

**Authors:** Emin Cadar, Horatiu Miresan, Laura Ana-Maria Dragan, Ana-Maria Pesterau, Ionuț Mircioiu, Carolina Pascale, Rodica Sirbu, Cristina-Crenguta Albu, Lucia Bubulac, Claudia Florina Bogdan Andreescu, Adrian Cosmin Rosca

**Affiliations:** 1 Faculty of Pharmacy, “Ovidius” University of Constanta, Capitan Aviator Al. Serbanescu Street, No. 6, Campus, Corp C, 900470 Constanta, Romania; emin.cadar@gmail.com (E.C.); horamires@yahoo.com (H.M.); cosmin.rosca@univ-ovidius.ro (A.C.R.); 2 Organizing Institution for Doctoral University Studies of “Carol Davila”, Faculty of Pharmacy, University of Medicine and Pharmacy Bucharest, Dionisie Lupu Street, No. 37, Sector 2, 020021 Bucharest, Romania; ana-maria.pesterau@drd.umfcd.ro (A.-M.P.); carolina.pascale@drd.umfcd.ro (C.P.); 3 Faculty of Pharmacy, “Titu Maiorescu” University, 031593 Bucharest, Romania; ionutu@yahoo.com; 4 Department of Genetics, Faculty of Dentistry, “Carol Davila” University of Medicine and Pharmacy, 020021 Bucharest, Romania; cristina.albu@umfcd.ro; 5 Department of Family Medicine, Faculty of Medicine, “Carol Davila” University of Medicine and Pharmacy, 020021 Bucharest, Romania; lucia.bubulac@umfcd.ro; 6 Department of Speciality Disciplines, Faculty of Dental Medicine, “Titu Maiorescu” University, 031593 Bucharest, Romania; claudia.andreescu@prof.utm.ro

**Keywords:** biocomposites, jellyfish collagen, biopolymers, sustainable marine resources

## Abstract

The Black Sea offers underutilized marine bioresources with potential for the development of materials intended for wound healing and skin tissue repair. In this study, new composite biomaterials were created using collagen peptides extracted from the jellyfish *Rhizostoma pulmo* (JPC) and hydroalcoholic extracts from the seaweeds *Ulva lactuca* (ALGU) and *Enteromorpha intestinalis* (ALGE). The marine-derived ingredients and resulting biocomposites were physicochemically and rheologically characterized and evaluated for antioxidant, antimicrobial, and biological activities on cell cultures relevant to wound healing. Two hydrogel biocomposites, B1 (JPC–ALGU 20%) and B2 (JPC–ALGE 20%), exhibited antioxidant activity of 65.2% and 63.5%, respectively, in the DPPH assay at a concentration of 400 mg/mL, and values of 1.26 μg/mg AAE and 1.15 μg/mg AAE, respectively, in the reducing power assay at a concentration of 800 µg/mL. Antibacterial activity was investigated against several Gram-positive and Gram-negative bacteria. The strongest effects were observed against *Staphylococcus aureus*. In vitro tests conducted on fibroblast and keratinocyte cultures revealed concentration-dependent cellular responses, while scratch assays confirmed the formulations’ potential to promote cell migration. These results support further investigation of jellyfish collagen–green algae biocomposites for wound-healing and skin tissue-repair applications, although additional dose optimization and in vivo validation are required.

## 1. Introduction

Natural biomaterials derived from the Black Sea remain relatively few; although there are multiple resources of marine bio-compounds with high potential for biomedical applications, these are little studied. However, there have been several interesting studies that have drawn attention to some biomaterials containing natural bio-compounds from marine resources, namely collagen extracted from marine resources and a series of valuable bio-compounds extracted from algae that may have applications in the medical field [1,2]. Currently, wound healing and tissue regeneration represent major challenges in human health, especially in the case of chronic wounds that do not follow the four normal stages of the healing process: they can become stuck in one or more of these stages due to several local and systemic factors [3]. Persistent inflammation, oxidative stress caused by ROS appearing in the wound, and microbial infection can cause additional tissue damage and prolong wound healing, and chronic wounds that are not effectively treated and overlap with other chronic diseases lead to increased morbidity [3,4]. In this context, alongside conventional medical therapies, alternative treatments based on natural compounds have been increasingly investigated for wound healing. Bioactive compounds derived from marine sources—such as collagen and active bio-compounds obtained from seaweed—have emerged as promising candidates for the development of materials for wound healing and tissue regeneration, owing to their biochemical compositions and biological properties as demonstrated by numerous scientific studies.

Collagen derived from marine organisms has been extensively studied, reflecting its growing importance in biomedical applications [5]. The use of collagen from terrestrial animals has raised concerns about potential immunological and inflammatory reactions, as well as the risk of disease transmission from animal-derived materials [6]. These limitations, along with regulatory, ethical, cultural, and religious constraints, have spurred research into safer and more acceptable alternative sources [7]. Various studies have been conducted to demonstrate the biocompatibility of marine collagen and highlight its role in skin and tissue regeneration [8,9,10]. Its potential role in wound healing is particularly relevant, as collagen-based biomaterials can support tissue regeneration and can be incorporated into dressings, hydrogels, and other systems designed for skin repair [11,12,13,14]. In addition to collagen derived from marine vertebrates, marine invertebrates represent another important source of collagen that, although less extensively studied, may also be relevant to wound-healing applications. Collagen has also been identified in the basement membranes of invertebrates [15,16,17]. Marine invertebrates represent an abundant but relatively unexplored source of collagen [18,19]. Interest in marine collagen derived from invertebrates has grown considerably, particularly due to studies addressing extraction methods, structural characterization, and the potential biomedical applications of collagen [20,21,22]. Among these organisms, jellyfish in particular have attracted increasing attention due to the structural and biological properties of their collagen and its potential application in biomedical materials [23,24,25]. The wound-healing potential of jellyfish collagen, extracted from *Rhopilema esculentum* and *Stomolophus meleagris*, has been studied by several researchers [26,27,28,29]. The *Rhizostoma pulmo* jellyfish is another species found in several seas and oceans, and in coastal waters, including the Black Sea, and its collagen has been studied by several researchers for its biomedical applications [30,31]. Studies conducted to identify the bioactive compounds in jellyfish compositions revealed high levels of collagen (rich in amino acids) alongside much lower levels of polysaccharides [12,21,32]. Due to its properties, jellyfish collagen, when combined with other bioactive compounds from various marine sources, exhibits enhanced antioxidant and antimicrobial activity and offers a good potential for wound healing [12,13,14].

Marine algae constitute an important marine resource for wound healing. They belong to various types and classes with differing compositions and are part of the marine plankton [33]. Seaweed extracts are rich in bioactive compounds, notably high levels of polysaccharides, as well as phenolic and flavonoid compounds, vitamins, and pigments, which impart significant biological properties; some of these are recognized as antioxidants and natural antimicrobial agents [34]. Studies have been reported involving the development of formulations based on jellyfish collagen and brown algae extract, or jellyfish collagen and extracts from the *Ganoderma lucidum* fungus species, which were investigated for medical applications [35,36].

The present study aims to valorize Black Sea marine bioresources, particularly collagen obtained from the jellyfish *Rhizostoma pulmo* and bioactive compounds extracted from the green algae *Ulva lactuca* and *Enteromorpha intestinalis* collected along the Romanian coast. Among the green algae investigated in this study, two macroalgal species, *Ulva lactuca* Linnaeus and *Enteromorpha intestinalis* (Linnaeus) Nees, were selected. Both species belong to the genus *Ulva*, family *Ulvaceae*, and phylum *Chlorophyta* [37,38].

The main objective was the development and evaluation of collagen and green algae extract composite biomaterials, intended for wound healing and skin tissue regeneration. Jellyfish collagen peptides (JPCs) and hydro-alcoholic green algae extracts (ALGU and ALGE) were first characterized individually; subsequently, various biomaterials were prepared yielding either hydrogels or freeze-dried powders—based on pure collagen or composite materials (collagen plus green algae)—in the form of membranes, porous matrices, and composite hydrogels.

The scientific novelty of the present study consists in the development and integrated evaluation of two new marine-derived hydrogel biocomposites, B1 (JPC–ALGU 20%) and B2 (JPC–ALGE 20%), combining *R. pulmo* collagen peptides with extracts of *U. lactuca* and *E. intestinalis*, respectively. Their physicochemical, rheological, antioxidant, and antimicrobial properties, as well as their in vitro effects on fibroblasts and keratinocytes, were evaluated to determine, for the first time, the potential of these new biocomposites for wound healing and skin regeneration.

## 2. Results

The results of the physicochemical analyses and biological characterization of the marine-derived materials are presented systematically in the following sections.

### 2.1. Biochemical Ingredients

The marine-derived materials used in this study are presented in Figure 1. *Rhizostoma pulmo* was processed to obtain a collagen hydrolysate extract, freeze-dried collagen, and freeze-dried collagen powder. The green algae *Ulva lactuca* and *Enteromorpha intestinalis* were collected from the Black Sea, processed, and used to obtain hydroalcoholic extracts. These collagen- and algae-derived materials were subsequently used for physicochemical characterization and for the preparation of the collagen–algae biocomposites.

### 2.2. Physicochemical Results of Marine Collagen Extract from Jellyfish

In order to establish the type of collagen and characterize the structure of collagen from the jellyfish *Rhizostoma pulmo*, we performed several types of physicochemical analyses.

The properties of jellyfish collagen were determined for its use in wound healing; other medical treatments for skin and mucosal regeneration are presented in Figure 2.

#### 2.2.1. SDS-PAGE Analysis

The SDS-PAGE profiles of collagen obtained using both extraction procedures were similar, as shown in Figure 2A. The presence of the characteristic α1 and α2 chains supported the identification of the extracted material as type I collagen.

#### 2.2.2. Melting Temperature of Jellyfish Collagen Determined by Differential Scanning Calorimetry (DSC)

The DSC analysis of collagen extracted from *Rhizostoma pulmo* showed a thermal denaturation temperature of 28.9 °C, as shown in Figure 2B.

#### 2.2.3. Circular Dichroism (CD) Spectral Analyses

The CD spectrum of *R. pulmo* collagen exhibited the characteristic spectral features associated with the collagen triple-helical conformation, with ellipticity peaks at approximately 220, 198, and 214.5 nm (Figure 2C).

#### 2.2.4. Thermogravimetric Analysis (TGA)

The TGA profile of *Rhizostoma pulmo* collagen showed two main stages of mass loss (Figure 2D). The first stage occurred between approximately 60 and 100 °C and accounted for 12.87% of the total sample mass. The second mass-loss stage began above 100 °C and became more pronounced between 280 and 400 °C. At temperatures above 600 °C, the residual mass represented 5.36% of the initial sample mass.

#### 2.2.5. FT-IR Analysis

Figure 2E shows the FT-IR spectrum of collagen extracted from the jellyfish *R. pulmo*. Analysis of the FT-IR spectrum reveals that amide A (at 3283 cm^−1^) exhibits N–H stretching vibrations, while amide B (at 2934 cm^−1^) exhibits CH_2_ vibrations. Absorption bands were also observed at 1647 cm^−1^, 1534 cm^−1^ and 1241 cm^−1^, corresponding to amide I, amide II, and amide III, respectively.

### 2.3. Study of Active Bio-Compounds Identified in Ingredients Derived from Black Sea Marine Resources

#### 2.3.1. Proximate Chemical Composition of Ingredients

The proximate chemical compositions of the *Rhizostoma pulmo* collagen-derived material and the hydroalcoholic extracts of *U. lactuca* and *E. intestinalis* are presented in Table 1. The PSC extraction procedure provided the highest collagen yield, and the proximate composition of the collagen peptides was therefore reported for the PSC-derived sample. Reference data for the *R. pulmo* collagen extract were obtained from Leone (2015) and Peșterău (2025), whereas reference data for the *U. lactuca* and *E. intestinalis* algal extracts were obtained from Cadar (2023) and Sirbu (2019) [20,35,39,40].

The analyzed materials showed distinct compositional profiles, with collagen representing 59.5 ± 0.4% DW of the jellyfish-derived material and carbohydrates accounting for 60.95 ± 2.02% and 61.56 ± 1.98% DW in *U. lactuca* and *E. intestinalis*, respectively. All measurements were expressed as the mean ± SD of three replicates. No statistically significant differences were observed for the analyzed parameters (*p* > 0.05).

#### 2.3.2. Amino Acid Content from Jellyfish Collagen Extract

The amino acid composition of collagen extracted from *Rhizostoma pulmo* is presented in Table 2. The data on the results were compared with the data reported for *Rhizostoma pulmo* specimens collected from the coast of Goa on the Arabian Sea by James (2023) and with specimens from the Romanian Black Sea coast collected by Peșterău (2025) [32,35].

Overall, the amino acid profiles were broadly comparable: glycine was the predominant amino acid (30.7%), followed by glutamic acid (14.8%), aspartic acid (9.5%), alanine (8.2%), and leucine (8.1%). For *Rhizostoma pulmo* samples from the Black Sea, the main amino acids therefore followed the descending order Gly > Glu > Asp > Ala > Leu > Lys > Arg. Cystine, serine, and histidine were detected at lower levels.

#### 2.3.3. Microscopic Studies for Collagen from the Jellyfish

Optical and electron microscopy analyses were performed in order to obtain further information on the structural organization of the collagen material. Figure 3A presents optical microscopy images of *Rhizostoma pulmo* jellyfish collagen recorded after 24, 48, and 72 h. The formation of complex fibrillar structures was observed over time, indicating that fibrillogenesis took place within the collagen matrix. Figure 3B shows the corresponding electron microscopy images. The collagen exhibited an irregular, dense, and fibrous morphology, with pores of variable size distributed throughout the material. Complex fibrillar features were also observed, including branched, ribbon-like filaments.

#### 2.3.4. Bio-Compounds from Seaweed

Other valuable ingredients obtained from marine resources include the green algae *Ulva lactuca* and *Enteromorpha intestinalis*. To ensure the safe use of these species as raw materials, particularly when harvested from marine ecosystems potentially exposed to contamination, their capacity to bioaccumulate heavy metals and other pollutants has been investigated.

##### UV-VIS Studies for Green Algae from Black Sea

The UV–VIS absorption spectra of *U. lactuca* and *E. intestinalis* are presented in Figure 4A and Figure 4B, respectively. UV–VIS spectral analyses were performed over the wavelength range of 300–1000 nm using acetone extracts of each algal species. The concentrations of the analyzed extracts were 17.5 mg/mL for *Ulva lactuca* and 12.7 mg/mL for *Enteromorpha intestinalis*. Both algal extracts exhibited characteristic absorption bands, indicating differences in their spectral profiles.

##### Total Phenolic and Flavonoid Content

The total phenolic content (TPC) and total flavonoid content (TFC) of the investigated materials are presented in Table 1. For *Ulva lactuca*, TPC was 418.50 ± 0.93 mg GAE/100 g DW and TFC was 18.6 ± 1.15 mg QCE/100 g DW. For *E. intestinalis*, the corresponding values were 415.62 ± 1.34 mg GAE/100 g DW and 15.1 ± 1.25 mg QCE/100 g DW, respectively. *U. lactuca* therefore exhibited slightly higher TPC and TFC values than *E. intestinalis*.

##### Individual Phenolic Content

The results for individual phenolic content are presented in Table 3. These results were obtained by HPLC analysis. All results are expressed in mg/100 g f.w. for *n* = 3 replicates. These results were compared with the data obtained by Sirbu (2020) [41].

#### 2.3.5. Vitamin Content

The vitamin content was analyzed for both types of algae: *Ulva lactuca* and *Enteromorpha intestinalis*. The results are presented as mean value ± SD for three measurements in Table 4. Results for the vitamin content for green algae from the Black Sea were previously reported by Cadar (2023) [39].

Among the vitamins identified, ascorbic acid was present at the highest concentration in both algal species and represented the predominant vitamin component of the investigated green algae [39].

### 2.4. Materials Obtained Based on Jellyfish Collagen from the Black Sea

Table 5 summarizes the main characteristics of the developed materials, including their color and physical appearance. These formulations include biomaterials based exclusively on collagen extracted from *R. pulmo*, as well as the B1 and B2 biocomposites obtained by combining jellyfish collagen with green algal extracts.

Figure 5 presents the biomaterials developed from jellyfish collagen, including collagen hydrogel, collagen lyophilisate, collagen powder, and collagen-based films. Composite films containing collagen and extracts of the green algae *Uva lactuca* or *Enteromorpha intestinalis* are also shown. In addition, Figure 5 includes collagen sponge dressings and porous matrices prepared either from jellyfish collagen alone or from collagen combined with *Ulva lactuca* and *Enteromorpha intestinalis* extracts. The two collagen–algae biocomposites, B1 and B2, are also presented.

### 2.5. Rheological Studies of Hydrogels B1 and B2

Rheological studies of hydrogels B1 and B2 describe the rheological behavior of the two new formulations. The rheological properties of the newly developed formulations, B1 and B2, were evaluated by analyzing the rheograms and flow curves of the hydrogel systems to assess their flow behavior and physical stability. The corresponding rheological profiles are presented in Figure 6.

Both collagen–algae biocomposites demonstrated pseudoplastic behavior throughout the investigated shear-rate range, with viscosity decreasing as the shear rate increased.

### 2.6. Biological Activities

#### 2.6.1. Antioxidant Activities

##### DPPH Test

Antioxidant activity, evaluated by the DPPH assay, was first studied for the following ingredients: jellyfish collagen peptides (JPC) presented in Figure 7A; extracts from *Ulva lactuca* (ALGU), and extracts from *Enteromorpha intestinalis* (ALGE), presented in Figure 7B; and biocomposites B1 and B2, presented in Figure 7C. The control was ascorbic acid. For example, the results obtained in the DPPH assay at a concentration of 400 µg/mL showed that the antioxidant activity was 65.2% for B1 and 63.5% for B2, compared to the results for the ingredients: 60.4% for ALGU, 59.5% for ALGE, and 27.6% for JPC. The IC_50_ values were also determined. The results showed that the IC_50_ value for JPC was 673.18 mg/mL, the IC_50_ for the *U. lactuca* extract was 283.15 mg/mL, and the IC_50_ for *E. intestinalis* was 295.60 mg/mL. Biocomposites B1 and B2 exhibited IC_50_ values of 254.30 and 268.40 mg/mL, respectively. The ascorbic acid standard exhibited an IC_50_ value of 272.30 mg/mL. Statistical analyses were performed using one-way ANOVA and pairwise *t*-tests. Statistically significant differences between JPC and the ascorbic acid reference standard are indicated by an asterisk (*) (*p* < 0.05).

##### Reducing Power Assay

The reducing power antioxidant activity of jellyfish collagen peptides (JPC), *Ulva lactuca* extract (ALGU), *Enteromorpha intestinalis* extract (ALGE), and the B1 and B2 biocomposites is presented in Figure 8.

##### Reducing Power Assay

For example, regarding the results obtained in the reducing power assay at a concentration of 800 µg/mL, the antioxidant capacity was 1.26 µg/mg AAE for B1 and 1.15 µg/mg AAE for B2. The corresponding values for ingredients were 0.521 µg/mg AAE for JPC, 0.98 µg/mg AAE for ALGU, and 0.91 µg/mg AAE for ALGE. The ascorbic acid reference standard showed a value of 1.962 µg/mg AAE. Among the investigated marine-derived samples, B1 exhibited the highest reducing power, followed by B2.

#### 2.6.2. Antimicrobial Activities

The antimicrobial activity of jellyfish collagen peptides (JPC), green algal extracts, and the B1 and B2 biocomposites was evaluated against two Gram-positive bacteria, *S. aureus* (ATCC 25923) and *S. epidermidis* (ATCC 12228); Gram-negative bacteria, *K. pneumoniae* (ATCC 13883), *P. mirabilis* (ATCC 25933), *E. coli* (ATCC 25922), and *P. aeruginosa* (ATCC 27853); and the fungal strain *C. albicans* (ATCC 10231).

Figure 9 shows the antimicrobial activity of the B1 and B2 biocomposites and jellyfish collagen peptides (JPC) against three microorganisms: *S. aureus* (Gram-positive), *P. aeruginosa* (Gram-negative), and *C. albicans* (fungal strain). Untreated microbial cultures served as negative controls. The strongest antimicrobial activity was observed against *S. aureus*. A slightly lower but comparable effect was observed against *P. aeruginosa*, whereas the weakest activity was recorded against *C. albicans*. As shown in Figure 9, B2 exhibited greater antimicrobial activity than B1. Both biocomposites produced greater inhibitory effects than JPC alone.

Figure 10 summarizes the inhibition-zone diameters obtained for the individual marine-derived ingredients and the B1 and B2 biocomposites. Chloramphenicol was used as the positive control. The inhibition-zone results confirmed the highest susceptibility of *S. aureus* and the lowest susceptibility of *P. mirabilis*.

The minimum inhibitory concentration (MIC) values are presented in Table 6. *S. aureus* showed the lowest MIC value (25 µg/mL), followed by *S. epidermidis* (50 µg/mL). MIC values of 75 µg/mL were recorded for *K. pneumoniae* and *E. coli*, whereas *P. mirabilis* showed MIC values above 100 µg/mL. The same MIC endpoint was recorded for B2 for each bacterial strain under the experimental conditions used.

Overall, the bioactive constituents of the green algal extracts contributed substantially to the antimicrobial effects of the two newly developed biocomposites, B1 and B2.

### 2.7. Biological Evaluation of New Composites B1 and B2 for Wound-Healing Applications

The use of fibroblasts and keratinocytes as complementary in vitro models is relevant for the preliminary evaluation of cellular responses associated with wound healing and skin regeneration. Biological studies using BALB/3T3 fibroblasts, clone A31, and human HaCaT keratinocytes were conducted in accordance with ISO 10993-1 (Biological Evaluation of Medical Devices—Part 1: General Requirements and Principles for the Evaluation of Biological Safety within a Risk Management Process, Geneva, Switzerland, 2025) [42].

#### 2.7.1. Biological Evaluations on BALB/3T3 Clone A31 Fibroblasts

The results of the cytotoxicity tests performed on fibroblasts from the BALB/3T3 cell line, clone A31, are presented in Figure 11. Cell viability testing was conducted in the range of 0.015–2 mg/mL. We observed that cell viability remained approximately constant at low concentrations. At 24 h, at a concentration of 2 mg/mL, cell viability slowly decreased from 94% to 75% for JPC, 68% for B1, and 65% for B2 (see Figure 11A).

Cell proliferation was tested after 48 h within the same concentration range of 0.015–2 mg/mL. It can be observed that, across the entire range, proliferation decreases progressively as concentrations increase. The decrease is slow and steady, reaching approximately 47–48% for B1 and B2 at concentrations of 2 mg/mL (see Figure 11B).

The scratch assay performed with BALB/3T3 fibroblasts from clone A31 is a widely used in vitro method for evaluating wound healing. The results of the scratch assay obtained for BALB/3T3 fibroblasts are presented in Figure 12. Wound closure was monitored over 48 h in untreated cells (CONTR) and in cells exposed to JPC, B1, and B2 (see Figure 12A).

After 48 h, the confluency rate reached 69% for B1 and 71% for B2 (see Figure 12B). Statistical analysis of the data was performed using one-way ANOVA and paired *t*-tests.

#### 2.7.2. Biological Evaluations in the Wound-Healing Process on HaCaT Cells

The cytotoxicity of compounds JPC, B1, and B2 was also evaluated in the human keratinocyte cell line HaCaT (Figure 13). After 24 h of incubation, cell viability remained high, with values of 94.5% for B1 and 95.5% for B2. After 48 h, cell proliferation decreased progressively with increasing sample concentration. At the highest concentration tested, 2 mg/mL, proliferation reached 41% for B1 and 39% for B2. Statistical analysis was performed using one-way ANOVA and paired *t*-tests.

The scratch test results obtained for HaCaT keratinocytes are shown in Figure 14A. The scratch area decreased progressively over the 48 h observation period, particularly in the case of cells treated with B1 and B2. Both biocomposites exhibited better wound healing than JPC used alone and than CTRL (represented by untreated samples). After 48 h, the confluence rate reached 78% for B1 and 79% for B2 in keratinocyte-derived cell lines.

## 3. Discussions

### 3.1. The Wound-Healing Process and Wound-Care Treatments

Wound healing is a complex biological process involving four partially overlapping phases: hemostasis, inflammation, proliferation, and tissue remodeling [43,44,45]. Hemostasis occurs immediately after injury and involves clot formation, primarily through the conversion of fibrinogen to fibrin [46,47,48]. The inflammatory phase was initiated shortly after injury and involves the activation of immune and cellular responses that contribute to pathogen defense and subsequent tissue repair [49,50,51]. During the proliferative phase, extracellular matrix (ECM) deposition, angiogenesis, fibroplasia, and epithelialization contribute to the formation of new tissue [52,53,54]. The remodeling phase involves the maturation and reorganization of the newly formed tissue, including the progressive replacement of type III collagen by type I collagen and the continuous turnover of ECM components [1,2,55]. Disruption or prolongation of one or more of these phases may impair the healing process and contribute to the development of chronic wounds [56,57,58,59]. Wound management therefore depends on factors such as wound type, location, depth, and the presence of infection, while chronic wounds may also require treatment of the underlying systemic or local conditions that interfere with tissue repair [60,61,62,63]. In addition to conventional wound-care approaches, increasing attention has been directed toward natural compounds derived from terrestrial plants and marine organisms because of their potential biological activities relevant to wound management [64,65,66]. The use of marine collagen in wound-healing treatments has been scientifically substantiated (due to an amino acid-rich composition, as well as the presence of minor amounts of polysaccharides and phenolic compounds); the substance is recognized as a potential therapeutic agent for wound healing that stimulates cell proliferation and migration, enhances angiogenesis, and exerts anti-inflammatory effects [12,67,68]. Collagen plays a role in all stages of the wound-healing process [69,70]. Microbial activity plays a significant role in the wound-healing process; bacteria can proliferate and subsequently contaminate, colonize, and infect a wound exposed to various microorganisms. The injured patient often cannot single-handedly combat the bacterial infections and oxidative stress that arise within the wound as early as the inflammatory phase. There is a need for bio-compounds with antibacterial and antioxidant properties—integrated into formulations possessing similar potential—to facilitate wound healing. Marine algae contain such bio-compounds (such as polysaccharides, polyphenols and flavonoids). Some of these have been investigated by other researchers for their biological roles in wound healing—specifically as standalone antimicrobial agents [71]. Among marine-derived biomaterials, collagen and algal bioactive compounds have attracted interest for their potential roles in supporting tissue-repair and wound-healing applications. In this context, the present study investigated collagen peptides obtained from the jellyfish *Rhizostoma pulmo* and bioactive extracts from two strains of green algae collected from the Romanian Black Sea coast. Based on the properties of these marine-derived materials, they were incorporated into hydrogel formulations B1 and B2, developed as novel biocomposites for potential wound-healing applications.

### 3.2. Physicochemical Characterization of Collagen Extracted from the Jellyfish

SDS-PAGE analysis provided information on the molecular organization of the collagen extracted from *Rhizostoma pulmo* (Figure 2A). The presence of distinct α1 and α2 chains is characteristic of type I collagen and is consistent with electrophoretic profiles previously reported for jellyfish-derived collagens [6,21,31]. These findings support the identification of the extracted material as type I collagen and indicate the preservation of its characteristic molecular organization during extraction.

DSC analysis showed a thermal denaturation temperature of 28.9 °C for *R. pulmo* collagen (Figure 2B). This value is lower than that reported for collagen derived from terrestrial animals (37 °C) by Subhan (2015), which is consistent with the generally lower thermal stability reported for marine collagen [72]. Similar thermal behavior has also been described for jellyfish collagen by Addad [31]. A lower thermal denaturation temperature for hydrogels can be highly beneficial for human tissues, particularly in biomedical applications such as tissue engineering, controlled drug delivery, and smart wound dressings. It contributes to in vivo tissue remodeling and may have direct implications for tissue healing and regeneration processes. Sumiyoshi et al. demonstrated that jellyfish collagen is suitable for topical application due to its rapid denaturation at body temperature, thereby minimizing the risk of potential unexpected immune reactions [73]. The CD spectrum (Figure 2C) with a positive ellipticity maximum at ~220 nm and a minimum (negative ellipticity) at ~198 nm provided further evidence for the preservation of the characteristic triple-helical conformation of the extracted collagen, in agreement with previous reports [72,73,74]. Recently, organic solvents such as ethanol have begun to be used to produce collagen-based biomaterials for biological applications (e.g., wound healing); therefore, it is necessary to understand collagen’s behavior in the presence of ethanol at both low (≤50%, *v*/*v*) and high (≥90%, *v*/*v*) concentrations. Gopinath et al. studied the influence of ethyl alcohol on the jellyfish collagen structure. CD ellipticity at 222 nm, characteristic of the triple-helical structure, does not correlate with the thermal stability of collagen [75]. These results indicate that when ethanol is used to process collagen-based biomaterials (intended for wound healing), factors such as temperature and duration should be taken into account to prevent extensive damage to the collagen triple-helical structure [75]. Thermogravimetric analysis (TGA) is important for collagen because it measures mass changes as a function of temperature, providing information on thermal stability, moisture content, and composition. TGA of *R. pulmo* jellyfish collagen revealed a two-stage mass-loss profile (Figure 2D). The initial mass loss, occurring around 100 °C, can be attributed to the removal of bound water, while the subsequent degradation, taking place between 280 and 400 °C, corresponds to the thermal decomposition of organic components. The residual ash content at temperatures above 600 °C was 5.36%. This thermal profile is consistent with data previously reported for collagen from other types of jellyfish [76]. The FT-IR spectrum (Figure 2E) exhibited the characteristic absorption bands associated with collagen, supporting the preservation of its molecular structure and triple-helical organization. Similar FT-IR characteristics have been reported for *R. pulmo* jellyfish collagen by James [32]. Taken together, the electrophoretic, spectroscopic, and thermal analyses provide complementary evidence supporting the identification of the extracted material as type I marine collagen and demonstrate the preservation of its characteristic structural organization.

### 3.3. Data on Bioactive Compounds from Individual Ingredients Extracted from Black Sea Marine Resources

Of the compounds presented in Table 1, the following discussion focuses on those most relevant to the biological performance and potential wound-healing applications of the developed marine-derived biomaterials.

Among the bioactive components obtained from the *Rhizostoma pulmo* jellyfish harvested from the Black Sea, the derived collagen accounts for a significant percentage of the total protein content and represents an element of major interest for the development of biomaterials intended for tissue repair. The results obtained in this study regarding protein and collagen content were comparable to data previously reported for *R. pulmo* by Pesterau et al. [35]. In addition to jellyfish-derived collagen, proteins were also identified in the green algae *Ulva lactuca* and *Enteromorpha intestinalis*, a finding consistent with previously published data [34,39,40]. From a biomaterials perspective, marine collagen can provide a structural component capable of contributing to all phases of the healing process [67]. This characteristic is particularly relevant for wound-healing applications, where the interaction between the biomaterial and the regenerating tissue may depend on the material’s ability to provide a suitable structural environment for cell adhesion and migration, as well as for tissue repair [55,56,57,58]. Collagen has been considered an accelerator of chronic wound healing, playing an active role in tissue remodeling and re-epithelialization [27,68,73]. In the present study, 16 amino acids were identified in the jellyfish protein component (JPC): 13 essential and 3 non-essential. Amino acid analysis of collagen peptides obtained from *Rhizostoma pulmo* from the Black Sea confirmed the presence of the characteristic amino acid profile associated with collagen, with glycine being the predominant amino acid, followed by glutamic acid and aspartic acid. This profile was comparable to those reported in previous studies on jellyfish-derived collagen [32,35]. The amino acid composition is relevant to the biological and structural properties of collagen-based biomaterials, as it contributes to the organization and functionality of the collagen network. Together with its protein nature, the presence of amino acids associated with collagen characteristics supports the potential use of *Rhizostoma pulmo* collagen as a structural component in marine biomaterials designed for tissue repair and regeneration [12,32,77].

Polysaccharides represent a second class of bio-compounds important for their role in skin tissue healing. The two species of green algae investigated in this study were characterized by a high carbohydrate content, indicating their potential as important sources of bioactive polysaccharides. Previous studies have similarly reported the presence and biological relevance of polysaccharides in green algae [78,79,80,81]. Among these compounds, ulvan is one of the characteristic polysaccharides for green algae [82,83,84,85]. Ulvan and other marine polysaccharides have been associated with several biological activities relevant to wound healing, such as antioxidant, antimicrobial and immunomodulatory effects, reduction in bacterial adhesion, and stimulation of cell proliferation [82,83,85]. Their potential importance in wound-healing applications has also been highlighted in previous studies investigating polysaccharides from marine algae [84,85,86,87]. Polysaccharides have also been identified in materials derived from *Rhizostoma pulmo*, albeit in much smaller quantities, despite previous studies investigating their biological relevance in regeneration processes [12,88]. Polysaccharides derived from jellyfish have been linked to cell migration and tissue regeneration, and may also contribute to antioxidant antimicrobial and anti-inflammatory effects [35,88]. The simultaneous presence of collagen and polysaccharide components in marine-derived systems may offer advantages, as the biological activities associated with these two classes of bio-compounds can be enhanced [74,89].

Phenolic compounds and flavonoids. The presence of phenolic compounds and flavonoids in the green algal extracts represents another relevant characteristic of the investigated marine resources. The values determined in the present study are presented in the Results Section, while their comparison with previously published data is addressed in the Discussion [34,39,40,41]. Phenolic compounds and flavonoids have been extensively investigated in green algae because of their antioxidant and antimicrobial activities [90,91,92]. These properties are particularly relevant for biomaterials intended for wound management, as oxidative stress and microbial contamination may interfere with the normal progression of tissue repair. The analysis of individual phenolic compounds by HPLC further confirmed the presence of bioactive phenolic constituents in the algal extracts [93,94]. The phenolic profiles of *Ulva lactuca* and *Enteromorpha intestinalis*, together with their high carbohydrate content, may therefore contribute to the biological functionality of the final biomaterial, particularly through antioxidant and antimicrobial mechanisms [95,96].

Vitamins. The identification of vitamins in both green algal species further supports their potential value as sources of biologically active compounds. Vitamins may contribute to the overall antioxidant properties of algal-derived materials. In particular, the presence of vitamins C and E may be relevant to the protection of biological structures against oxidative damage. Previous studies have also highlighted the nutritional and bioactive potential of *Ulva lactuca* and other green algae [97,98,99]. Furthermore, studies conducted on certain green algae collected from the Romanian Black Sea coast have demonstrated the presence of biologically active compounds and their potential—through various mechanisms, particularly biological ones—to accumulate heavy metals and act as depolluting agents [100,101].

Microscopic investigations further provided information regarding the structural organization of the marine collagen. The characteristics observed via optical and electron microscopy in the present study, along with their detailed comparison with data from previous studies, attest to a homogeneous porous organization [102]. The fibrillar and porous organization we observed is relevant for future applications in which the collagenous structures within these biomaterials will serve as interconnected networks capable of facilitating cell infiltration, adhesion, and proliferation [102,103].

Overall, the marine resources investigated from the Black Sea provide complementary classes of compounds that can contribute to the development of functional biomaterials for wound healing. Collagen derived from *Rhizostoma pulmo* can provide a structural protein network, while algal polysaccharides can help support intense biological activity (antioxidant and antimicrobial) and facilitate interactions for the repair of damaged tissue. Concurrently, phenolic compounds, flavonoids, and vitamins can enhance the antioxidant and antimicrobial potential of the formulations. The amino acid-rich composition and structural organization of marine collagen can contribute to tissue remodeling. Taken together, these complementary properties drove the decision to combine jellyfish-derived collagen with green algae extracts to develop final marine biocomposite formulations intended for potential wound-healing applications.

### 3.4. Biomaterials Made from Jellyfish Collagen and Green Algae from the Black Sea

In our study, one of the main ingredients consisted of collagen hydrogels or freeze-dried collagen powders prepared from collagen extracted from the jellyfish *Rhizostoma pulmo*.

Green algae extracts constituted the second main component. A 70% ethanol solution was selected for the extractions because, compared to other ethanol concentrations (50% or 96%), it yielded a wider range of bioactive compounds. Furthermore, hydroethanolic extracts proved to be richer in various classes of bioactive compounds than aqueous or ethereal extracts, as demonstrated in our previous studies [104]. The new biocomposites can be produced as lamellar films, sponge-like dressings, porous matrices, and composite hydrogels derived from hydroethanolic green algae extracts and jellyfish collagen, as illustrated in Figure 5 and described in Section 4. Among the pharmaceutical forms potentially relevant for wound care are porous matrices, biodegradable films, and hydrogels. These collagen-based biomaterials offer favorable biodegradability and potential applications in the management of acute and chronic wounds, supported by the complementary roles of collagen and green algae compounds during the various stages of wound healing. Potential biomedical applications include biodegradable dressings, hydrogels for wound care, formulations for diabetic foot ulcers, and materials designed to treat burns, other skin lesions, and postoperative wounds. However, further studies—physicochemical, biological, preclinical in vivo, and clinical—are required to confirm their safety, stability, and therapeutic efficacy prior to potential clinical use.

#### Hydrogel Formulations Based on Jellyfish Collagen and Green Algae Extracts

Two new hydrogel-based biocomposites, designated B1 and B2, were developed using marine-derived compounds extracted from *R. pulmo*, *Ulva lactuca*, and *Enteromorpha intestinalis.* Their biological properties were evaluated to determine their potential relevance to the wound-healing process. Rheological studies were performed to evaluate the homogeneity, flow behavior, and physical stability of the formulations. Rheograms and flow curves indicated that both hydrogels exhibited pseudoplastic behavior, with a hysteresis loop between the ascending and descending shear-rate cycles. This rheological profile suggests thixotropic behavior that may contribute to the physical stability and practical application of the formulations. Similar rheological characteristics have been reported for pharmaceutical systems based on marine collagen combined with other seaweed-derived materials [35,36,105].

### 3.5. The Contribution of New Composites B1 and B2 and Their Ingredients in the Wound-Healing Process

#### 3.5.1. Antioxidant Activities

Antioxidant properties are important for biomaterials intended for wound healing, as they help maintain a cellular environment conducive to tissue repair. Limiting reactive oxygen species (ROS) and reducing oxidative stress at the wound site require the significant action of antioxidant compounds. The antioxidant activity of the new formulations, B1 and B2, is supported by their constituent ingredients but may also result from the synergistic potentiation of those ingredients. Antioxidant activity was evaluated using two methods that differ in their mechanisms of action yet yield conclusive results. In the DPPH assay, formulations B1 and B2 exhibited higher antioxidant activity than JPC and the algal extracts ALGU and ALGE as concentrations increased. The IC_50_ values for B1 and B2 were lower than those of the corresponding individual ingredients. Similarly, in the assay measuring reducing capacity, B1 demonstrated the highest reducing capacity, followed by B2 and the corresponding ingredients. Antioxidant activity can be correlated with the diversity of bioactive constituents. Green macroalgae are characterized by the presence of phenolic compounds, flavonoids, polysaccharides, vitamins, and other metabolites with antioxidant properties. The contribution of phenolic compounds to the antioxidant activity of green algae has been reported by several researchers [92,95,96,106,107]. Jellyfish-derived collagen contains peptides, amino acid sequences, and smaller amounts of polysaccharides [12,21,108,109,110]. The antioxidant activity of collagen peptides derived from jellyfish has been demonstrated by several authors [111,112,113]. Similar evaluations using DPPH and reducing power assays were reported by Pesterau (2025) and Cadar (2026), whose findings are consistent with the antioxidant behavior observed in the present study [35,74].

Importantly, although B1 and B2 exhibited greater antioxidant activity than their individual components, the observed effects may result from additive, synergistic, or antagonistic interactions between collagen-derived peptides and algal polyphenols, polysaccharides, and other bioactive constituents. The present experimental design was not specifically intended to distinguish among these types of interactions; therefore, the greater antioxidant activity of the combined formulations should not be interpreted as evidence of a specific interaction mechanism. Further studies using appropriate combination or interaction models are required to determine the nature of these interactions. Moreover, because the DPPH and reducing power assays are chemical antioxidant assays, the potential contribution of these antioxidant properties to wound healing requires further confirmation in biological systems and in vivo wound models.

#### 3.5.2. Antimicrobial Activities

Antimicrobial activity is particularly relevant to wound-healing applications, as microbial colonization and infection can prolong inflammation, delay tissue repair, and increase the risk of wound-related complications. In the present study, the individual marine-derived ingredients and the B1 and B2 biocomposites exhibited antimicrobial activity against Gram-positive and Gram-negative bacterial strains, as well as against *C. albicans*, although the extent of the response varied among the investigated microorganisms. Among the bacterial strains, *S. aureus* exhibited the highest susceptibility, whereas *P. mirabilis* was the least susceptible. Inhibitory activity was also observed against *P. aeruginosa* and *E. coli*, while the response of *C. albicans* was comparatively lower. Overall, B2 produced slightly greater inhibition than B1, and both biocomposites showed greater inhibitory effects than their individual components. The antimicrobial activity of the biocomposites may be related to the contribution of several bioactive compounds, including polysaccharides, phenolic compounds, and other secondary metabolites derived from green algae, as well as collagen-derived peptides. Differences in bacterial susceptibility may partly reflect differences in bacterial cell-wall structure, as the outer membrane of Gram-negative bacteria can limit the penetration of certain antimicrobial compounds. However, the observed susceptibility of *P. aeruginosa* and *E. coli* indicates that the antimicrobial response may depend on both the microorganism and the composition of the investigated material. Similar antimicrobial effects of green algal extracts have been reported by Alghazeer (2013) and Anjali (2019) [114,115].

As B2 showed the most pronounced antimicrobial activity under the investigated conditions, minimum inhibitory concentration (MIC) testing was performed for this biocomposite and its individual components, JPC and ALGE. Antimicrobial activity of composite materials based on collagen- and other marine-derived compounds has also been reported in previous studies [35,74,105].

#### 3.5.3. In Vitro Studies on Cell Lines

Within the skin, keratinocytes, melanocytes, and fibroblasts play essential roles in maintaining tissue structure and function; they also play a significant role in the wound-healing process [116,117]. The use of fibroblast and keratinocyte cell lines for in vitro testing is necessary to obtain primary data for evaluating cellular responses relevant to skin repair. Their significant proliferative capacity and involvement in cell migration make them particularly suitable for preliminary wound-healing studies.

The in vitro biological activity of biocomposites B1 and B2 and jellyfish collagen peptides (JPC) was evaluated in comparison with untreated control samples (CTRL). The biological response to JPC and biocomposites B1 and B2 was concentration-dependent. For BALB/3T3 fibroblasts, cell viability remained relatively high across the entire concentration range studied, whereas cell proliferation decreased, reaching 47–48% for B1 and B2 at a concentration of 2 mg/mL. In the case of HaCaT keratinocytes, proliferation also decreased, reaching 41% for B1 and 39% for B2 at 2 mg/mL. Scratch assays demonstrated cell migration and progressive wound closure in both BALB/3T3 fibroblast and HaCaT keratinocyte cultures.

The scratch-assay findings indicated that both B1 and B2 promoted greater wound closure than JPC alone and the untreated control, suggesting that the incorporation of green algal extracts into the collagen-based formulations may contribute to enhanced cell migration. Under the experimental conditions used, B1 and B2 demonstrated promising effects on cell migration relevant to tissue repair. Our results were confirmed by a comparison with similar in vitro approaches using fibroblast and keratinocyte cultures, including scratch assays and cytotoxicity assessments, which have been reported in previous studies on wound healing [12,118,119,120,121]. These findings highlight that, regarding the biocompatibility of the investigated formulations, further research involving in vivo testing is essential to harness their potential for wound-healing applications. Taken together, the antioxidant and antimicrobial properties of materials B1 and B2, along with the cellular migration observed at the concentration used in the scratch assay, support further research into their potential use as biomaterials for wound healing. However, these results must be interpreted in the context of future studies, as in vivo wound-healing tests and clinical trials are required.

## 4. Materials and Methods

To develop novel marine-derived pharmaceutical systems for wound healing and tissue regeneration, the jellyfish *Rhizostoma pulmo* and the green algae *Ulva lactuca* and *Enteromorpha intestinalis* were selected as the main biological raw materials. Both algal species were collected from the Romanian Black Sea coast, from the Costinești and Mangalia areas. The jellyfish samples were collected from two distinct sites along the Romanian Black Sea: waters near the Mamaia area and a sheltered area within the Port of Constanța. The collected biological materials were subsequently used for the isolation of bioactive compounds, physicochemical characterization, and development of pharmaceutical formulations with potential applications in wound healing and tissue regeneration.

### 4.1. Chemical Reagents

All reagents and chemicals used in this study were of analytical grade and were selected according to the requirements of each experimental procedure. The reagents used were purchased from Bio-Rad Laboratories (Hercules, CA, USA) for SDS-PAGE analysis and from Merck KGaA (Darmstadt, Germany) for glacial acetic acid (99–100%). The microbial strains and culture media used for the antimicrobial assays were provided by the Sanitary Veterinary and Food Safety Directorate of Constanța, Romania. Biocompatibility studies were performed using the human keratinocyte cell line HaCaT, provided by Cell Systems GmbH (Troisdorf, Germany), and the murine fibroblast cell line BALB/3T3, obtained from the American Type Culture Collection (ATCC, Manassas, VA, USA). The two cell lines were maintained in DMEM supplemented with 1% penicillin–streptomycin and 10% fetal bovine serum (FBS). All cell-culture reagents were supplied by Thermo Fisher Scientific (Waltham, MA, USA). Additional chemicals, analytical standards, and laboratory consumables were obtained from Sigma-Aldrich, Merck KGaA (Darmstadt, Germany).

### 4.2. Preparation of Marine Extracts

#### 4.2.1. Green Algae

The green algae *Ulva lactuca* and *Enteromorpha intestinalis* were collected and transported in unused plastic bags containing seawater to minimize moisture loss during transport. To remove adhering salts, sand and other impurities, the algal material was carefully washed, first with drinking water and subsequently with double-distilled water. After cleaning, the algae were dried in the dark and ground into a powder using an electric grinder. The resulting powders were passed through a 3 mm mesh sieve to obtain a more uniform particle size. Ethanol–water mixtures at concentrations of 50%, 70%, or 90% (*v*/*v*) were considered as extraction solvents. Based on our previous studies, 70% ethanol was selected because it provided the most suitable extraction of the compounds of interest for the purposes of the present study [104]. The algae powders were macerated for 24 h at 23 ± 1 °C. The extraction procedure was performed in hermetically sealed glass or amber containers. The new mixtures were periodically shaken during maceration to promote solvent extraction of the bioactive compounds. The resulting solutions were concentrated using a rotary evaporator. Successive filtrations were performed to obtain a clear solution. Trace amounts of residual ethanol may remain in the algal extracts following the hydroalcoholic extraction process. The green algae extracts were stored in a refrigerator and kept for further analysis [101].

#### 4.2.2. Obtaining Collagen and Collagen Peptide Extracts from the Jellyfish

The extraction procedures have been previously validated using hydroxyproline content as an indicator of collagen recovery, according to a method developed by Sirbu (2019) and Pesterau (2024) [122,123]. The collagen extraction from the Black Sea *Rhizostoma pulmo* jellyfish began with a pretreatment step. The jellyfish material was cleaned by successive washing with ultrapure water and sodium chloride solutions, followed by demineralization using 0.5 M EDTA-Na supplied by Sigma-Aldrich, Merck KGaA (Darmstadt, Germany).

Collagen was extracted using a validated method using the ASC and PSC processes. Collagen extraction was performed according to the protocol described by Cadar et al. (2024) for the isolation of collagen from marine invertebrates [5]. The collagen extracts obtained were in the form of collagen hydrolysate, lyophilized collagen and lyophilized powder.

The jellyfish-derived material was fragmented and washed for 30 min. For the ASC procedure, the pre-treated material was immersed in 0.5 M acetic acid supplied by Merck KGaA (Darmstadt, Germany). For the PSC procedure, following the washing step, a 10% (*w*/*v*) pepsin solution from Sigma-Aldrich, Merck KGaA (Darmstadt, Germany) was added. Both mixtures were maintained at 4 °C. After three days, the suspensions were centrifuged at 10,000 rpm for 60 min. From the collected supernatants, collagen was precipitated using a 2 M NaCl solution containing 0.05 M Tris buffer (supplied by REMED Prodimpex, Pantelimon, Romania), yielding solutions that were subsequently adjusted to pH 7.0. Following another centrifugation step at 20,000 rpm for 60 min, the collagen precipitates were recovered and resuspended in an acidic solution (0.5 M acetic acid). Subsequently, the acidic solutions underwent dialysis for 2 h for purification purposes. The extraction and purification steps were carried out at 4 °C. The collagen extraction yield was calculated with the following formula:

(1) $$Collagen\;yield\;\left(wet\right)\%=\frac{Weight\;of\;extracted\;collagen\;(g)}{Weight\;of\;wet\;jellyfish\;(g)}\;\times \;100$$

Collagen peptides were obtained from *Rhizostoma pulmo* using the enzymatic hydrolysis method described by Felician et al. (2019) [26]. The solution was prepared in a thermostatically controlled water bath by dispersing 1.5 g of previously extracted collagen in 250 mL of water. Collagenase type II was added at 37 °C, and stirring of the mixture was continued for 5 h. The enzymatic activity of the mixture was terminated by heating at 95 °C for 10 min. Then the mixture was cooled to 24 °C and centrifuged at 5000 rpm for 30 min. Jellyfish collagen peptides (JPC) were collected from supernatant and stored at 4 °C until further use. A part of the peptide-containing preparation was retained in hydrogel form for subsequent formulation studies. The remaining material was lyophilized to obtain either intact matrices or collagen peptide powder after grinding. Lyophilization was performed using a Labconco freeze-dryer (Labconco Corporation, Kansas City, MO, USA) equipped with Lyo-Works™ operating Software/Firmware Update Procedure was CleanWorks OS software version 10688001, Application Software: 10688001 G., Product(s): SteamScrubber, FlaskScrubber and FlaskScrubber Vantage Laboratory Glassware Washers. for monitoring and controlling the temperature, vacuum level, and data acquisition.

### 4.3. Physicochemical Analysis of Jellyfish Rhizostoma pulmo

#### 4.3.1. SDS-PAGE Analysis

The collagen type of jellyfish was characterized using SDS-PAGE analysis; for sample preparation, to remove insoluble components, 15 g of biological material was centrifuged at 24 °C for 5 min. The resulting supernatant was diluted with phosphate-buffered solution and subsequently mixed with Laemmli buffer solution. The samples were heated gradually for 5 min. at 90 °C and 95 °C to ensure complete denaturation of the proteins. The Laemmli buffer solution facilitated protein solubilization before electrophoretic separation. Electrophoresis was performed for 30 min on polyacrylamide gels at an initial voltage of 85 V. The applied voltage was then increased to 95 V and maintained until protein migration was complete. Following electrophoresis, the gels were stained overnight to visualize the separated protein bands. A GD-800 densitometer (from Bio-Rad Laboratories, Hercules, CA, USA) was used for the densitometric analysis of the protein bands. Gel images were processed and analyzed using Quantity 1 software, version 4.6.3 (from Bio-Rad Laboratories, Hercules, CA, USA). The SDS-PAGE procedure was adapted from previously reported protocols for the characterization of proteins and collagen derived from marine organisms [21,31,32].

#### 4.3.2. Determination of the Melting Temperature of Collagen by Differential Scanning Calorimetry (DSC)

The denaturation temperature (Td) of collagen extracted from *Rhizostoma pulmo* was determined using Differential Scanning Calorimetry (DSC), which allows for monitoring of temperature-induced conformational changes in proteins. The equipment for the measurements was a Micro DSC VII (Setaram, Lyon, France). It possesses a temperature control system for gradual and controlled heating of the samples. The thermal development was monitored over time and the temperature was progressively increased at a heating rate of approximately 1 °C/min. The denaturation temperature was defined as the temperature corresponding to the irreversible disruption of the triple-helical structure of collagen, indicated by changes in a characteristic signal. All analyses were performed in triplicate.

#### 4.3.3. Circular Dichroism Spectral Analysis (UV-CD)

The structural conformation of collagen extracted from *Rhizostoma pulmo* was investigated using circular dichroism (CD) spectroscopy, a technique sensitive to the secondary structure and molecular organization of biomacromolecules. The suitability of this method for characterizing collagen and other marine-derived proteins has been demonstrated in recent studies by Pesterau (2025) and Pascale (2026) [35,36]. The equipment utilized for the measurements was a Jasco J-810 spectropolarimeter (JASCO Corporation, Tokyo, Japan), and the spectra were acquired and processed using Spectra Manager software (JASCO Corporation, Tokyo, Japan). CD spectra were recorded over the wavelength range of 195–250 nm. The jellyfish collagen was dissolved in 0.5 M acetic acid before analysis.

#### 4.3.4. Thermogravimetric Characterization of Jellyfish Collagen

The thermal stability and degradation behavior of collagen extracted from the Black Sea *Rhizostoma pulmo* jellyfish were evaluated by thermogravimetric analysis (TGA). Measurements were performed using a TGA 8000 thermogravimetric analyzer equipped with an autosampler (PerkinElmer, Shelton, CT, USA). For each determination, approximately 10 mg of lyophilized collagen was placed in an alumina crucible. The sample was initially maintained at 25 °C for 10 min and subsequently heated from 25 to 600 °C under an inert nitrogen atmosphere. DTG and TGA profiles curves were obtained simultaneously. These profiles enabled the determination of the temperature corresponding to the maximum degradation rate (Tmax), the percentage mass loss during each degradation stage, and the residual mass remaining from analysis. Data were collected and processed using the software associated with the TGA 8000 system. The results indicated that the initial mass loss occurred around 100 °C. Degradation of the collagen protein took place, with the main thermal decomposition occurring between approximately 280 and 400 °C. The residual inorganic fraction, corresponding to the ash content, was 5.36% at the end of the thermal analysis. The calculation of the ash content of jellyfish collagen samples at the end of the analysis is done according to the equation:

(2) ash content (inorganic component) (%)=(total weight (%100))−(water (%)+organic component(%))

#### 4.3.5. Spectroscopic FT-IR Analysis

FT-IR spectra were recorded using a Jasco FT/IR-4200 spectrophotometer operated with Spectra Manager software (JASCO). The Jasco FT/IR-4200 spectrophotometer and its Spectra Manager software are manufactured by JASCO Corporation (heandquartered in Hachioji,Tokyo, Japan). For sample preparation, the collagen-derived materials were mixed with acetic acid at a 1:1 (*v*/*v*) ratio. For spectral acquisition, the samples were dried, finely ground, and homogenized with potassium bromide (KBr), after which the resulting mixtures were analyzed in the infrared region. Spectra were recorded over the range of 700 to 4000 cm^−1^, which was selected for the identification and characterization of the absorption bands associated with collagen peptides [38].

### 4.4. Analysis of the Biochemical Composition of Marine Studied Species

The moisture and ash contents of the analyzed samples were determined according to the standardized AOAC methods used for proximate analysis of biological materials [124]. For moisture determination, 2 g of each sample was dried in a thermostatically controlled oven at 105 °C until a constant mass was achieved. Moisture content was calculated from the difference between the initial and final sample masses and reported as a percentage. Ash content was quantified after incineration of the samples in a muffle furnace maintained for 4 h at 500 °C, or until a constant mineral residue was obtained [124].

#### 4.4.1. Biochemical Composition for Jellyfish from Black Sea

The experimental methods applied in this study were selected and adapted from existing ones in the literature for jellyfish compositions [12,20,21,22].

Determination of total protein content. The determination of total protein content was carried out according to the specific methods for jellyfish, described by De Domenico (2019) [21]. The measurements were taken using a system consisting of an Infinite^®^ M200 PRO Quad4 microplate reader (from Tecan Group Ltd., Männedorf, Switzerland). As the reference standard for construction of the calibration curve, BSA was used. All measurements were conducted in triplicate. The data are presented as percentages.

Determination of total lipid content. Total lipid content was determined using a gravimetric method adapted from the protocol described by Percy (1981), with a micro-Soxhlet extraction system [125]. For each determination, extraction was performed on a 100 mg sample using a chloroform–methanol mixture (2:1, *v*/*v*) to recover the lipid fraction. The organic solvent was subsequently evaporated. Total lipid content was then determined gravimetrically and expressed as a percentage of the sample mass.

Determination of carbohydrate content. The carbohydrate extraction protocol was adapted from the method of Zhang, with optimization of the principal experimental parameters [88]. Briefly, the samples were extracted with water for 4 h at 100 °C. Subsequently, polysaccharide fractions were separated by ethanol-induced precipitation and three ethanolic fractions of different final concentrations were obtained. All the fractions obtained under these conditions showed distinct physicochemical characteristics, demonstrating the influence of ethanol concentration on polysaccharide separation.

#### 4.4.2. Composition of Green Algae

Determination of total nitrogen and protein content. In the composition of the algal samples, total nitrogen content was determined using the Kjeldahl method. Analyses were performed with a UDK DK6 digestion system equipped with a UDK 127 distillation unit and dedicated VELP software (VELP Scientifica, Usmate Velate, Italy). Following digestion, the resulting solution was alkalized to convert ammonium ions into ammonia. The released ammonia was separated by steam distillation and collected in a boric acid solution. The amount of trapped ammonia was subsequently quantified by titration with standardized hydrochloric acid. The total nitrogen content was calculated from the titration data and subsequently converted into protein content using the appropriate nitrogen-to-protein conversion factor. The results were expressed as a percentage of the initial dry mass of the algal material, according to previously described methods [40,41].

Determination of total lipid content. In the composition of algal samples, total lipid content was determined using a Soxhlet extraction method adapted from the protocol described by Rohani-Ghadikolalel et al. (2012) [126]. Dichloroethane was used as the extraction solvent, and the process was carried out for 5 h to ensure efficient recovery of the lipid fraction from the algal material. After extraction, the solvent was removed by evaporation, and the remaining lipid residue was quantified gravimetrically. Two aliquots of each sample were analyzed, and the total lipid content was expressed as a percentage of the initial mass of the algal powder. The procedures were executed in conformity with previously methods reported by Ansari (2015) [127].

Total carbohydrate content of green algae. In the study of carbohydrate content, the phenol–sulfuric acid colorimetric method adapted by Albalasmeh (2013) [128]. In this assay, carbohydrates react with a 5% aqueous phenol solution under strongly acidic conditions to form a colored product whose intensity is proportional to the carbohydrate concentration. At 490 nm, the absorbance was read using a VWR UV-6300 PC dual-beam UV–VIS spectrophotometer (from VWR International, Radnor, PA, USA). The carbohydrate content was calculated based on the calibration curve made with glucose according to the equation:

(3) y = 0.1009x − 0.0024; R^2^ = 0.992

where x represents the absorbance and y the carbohydrate concentration, and the correlation coefficient R^2^ is 0.992. The results were expressed as percentages [123,128].

Evaluation of total dietary fiber. Total dietary fiber content, including the soluble and insoluble fiber fractions, was determined according to the methodology described by Yaich et al. (2015) [129]. Two complementary analytical approaches were applied: the Prosky gravimetric method and the Englyst enzymatic–chemical method. Compared with the Englyst method, the Prosky procedure offered a simpler experimental workflow and greater practical applicability for the routine determination of dietary fiber [129].

### 4.5. Determination of Amino Acid Content from Rhizostoma pulmo collagen

The profile of amino acid content of collagen isolated from marine jellyfish *Rhizostoma pulmo* was determined by high-performance liquid chromatography using HPLC equipment; a Waters 626 chromatographic system with a 474 fluorescence detector; a 717 Plus autosampler and a Millennium Chromatography Manager software (Milford, MA, USA, Waters Corporation). To prepare the samples, 0.2 g of lyophilized jellyfish collagen was homogenized with 5 mL of double-distilled water; 50 μL of DL-norleucine internal standard solution and 0.75 mL of trifluoroacetic acid (TFA). The homogenized mixture was incubated for 30 min, followed by centrifugation at 3500 rpm at 4 °C for 30 min. The supernatant was carefully collected, filtered, and concentrated to dryness under a light nitrogen stream. The resulting residue was subsequently reconstituted in distilled water (1 mL) prior to chromatographic analysis. Amino acid separation, derivatization, and quantification were performed according to the AccQ-Tag protocol supplied by Waters Corporation (Waters, Milford, MA, USA). The experimental procedure was adapted from the method described by Barzideh et al. (2014) for characterization of the amino acid composition of collagen obtained from other jellyfish species [108].

### 4.6. Microscopic Analysis of Rhizostoma pulmo Collagen

The morphological characterization of collagen isolated from jellyfish *Rhizostoma pulmo* was examined by optical microscopy to evaluate its structural organization and general appearance. Images were recorded after 24, 48, and 72 h, using a 20 µm scale. The analysis focused on the morphology of the collagen fibers, structural uniformity and any changes associated with the extraction and lyophilization processes. Optical microscopy was carried out using a Nikon Eclipse microscope which has a Nikon Digital Sight DS-Fi2 digital camera (Nikon Corporation, Tokyo, Japan). The collagen microstructure was further investigated by SEM technique (scanning electron microscopy). Before analysis, the JPC samples were covered with a thin gold coating prior to SEM analysis using an EM ACE600 sputter coater (Leica Microsystems,Weitzlar, Germany). The samples were examined using an SU7000 scanning electron microscope (Hitachi High-Tech Corporation, Tokyo, Japan) at different magnifications and an accelerating voltage of 3 kV.

### 4.7. Important Bioactive Compounds from Marine Algae

#### 4.7.1. UV–VIS Spectroscopic Analysis of Green Algae from the Black Sea

UV–VIS spectroscopy was used as a complementary method for the physicochemical characterization of the two Black Sea green algal species and to have new information regarding the main classes of bioactive compounds present in their extracts. Absorption spectra were obtained using a VWR UV-6300 PC UV–VIS double-beam spectrophotometer (VWR International, Radnor, PA, USA). Acetone extracts of *Ulva lactuca* and *Enteromorpha intestinalis* were analyzed over the wavelength range of 300–1000 nm. The concentrations of the analyzed extracts were 17.5 mg/mL for *Ulva lactuca* and 12.7 mg/mL for *Enteromorpha intestinalis*.

#### 4.7.2. TPC and TFC of Green Algae and of Jellyfish

##### Evaluation of Total Phenolic Content

To determine the total phenolic content (TPC) for both green algae extracts, the Folin–Ciocalteu colorimetric method was used, adapted to the particularities of each sample type. For *Ulva lactuca* and *Enteromorpha intestinalis* extracts, the determination was made following the modified protocol described by Cadar (2023) and Choudhary (2023) [39,106]. At 765 nm, the absorbance of the samples was read with a VWR UV-6300 PC, double-beam UV–VIS spectrophotometer (from VWR International Company, Radnor, PA, USA). For quantification of phenolic compounds from green algae, the following equation from the gallic acid calibration curve was used:

(4) y = 0.0078x + 0.1861;    R^2^ = 0.9959

All determinations were made in triplicate. The results were presented as the average of the three measurements and reported in mg GAE/100 g d.w.

The total phenolic content of collagen extracted from *Rhizostoma pulmo* jellyfish was determined using a slightly modified Folin–Ciocalteu colorimetric method, based on the protocols described by Leone (2019) and Stabili (2021) [110,111]. Briefly, 100 μL of the collagen sample was added to 500 μL of sodium carbonate solution and 500 μL aliquot of Folin–Ciocalteu reagent. The mixture was incubated for 2 h to allow for color development. Absorbance was subsequently measured using a VWR UV-6300 PC double-beam UV–VIS spectrophotometer (VWR International, Radnor, PA, USA). Quantification was performed based on a gallic acid calibration curve, according to the equation:

(5) y = 0.0067x − 0.029;    R^2^ = 0.9998

The results were reported in mg GAE/100 g d.w., facilitating the evaluation and comparison of the total phenolic compound content of jellyfish collagen with the data available in the literature [110,111].

##### Evaluation of Total Flavonoid Content

Quantification of total flavonoid content (TFC) was carried out using the colorimetric assay based on the formation of flavonoid–AlCl_3_ complexes. Quercetin was used as the reference standard, following the method described by Ghasemzadeh (2010) and subsequently adapted by Lukiati (2020) [130,131]. A quercetin stock solution with a concentration of 100 ppm was prepared using methanol to bring the volume to 10 mL. Working standard solutions at different concentrations were subsequently prepared from the stock solution. For color development, 0.5 mL of 10% AlCl_3_, 5 mL of methanol solution and 0.5 mL of 0.5 M potassium acetate were introduced into each standard solution. The solution was diluted to a final volume of 10 mL with distilled water. The mixtures were left to incubate for 30 min. at room temperature. At 431 nm, the absorbance was read on a VWR UV-6300 PC double-beam UV–VIS spectrophotometer (VWR International, Radnor, PA, USA). The TFC was calculated using the quercetin calibration curve:

(6) y = 0.0078x + 0.1842; R^2^ = 0.999

The results were expressed in mg quercetin equivalents per gram of seaweed (mg QCE/100 g).

##### Individual Phenolic Content

Individual phenolic compounds in the green algal extracts were identified and quantified by the HPLC method, following the protocol described by Goupy (1999) [131]. Analyses were performed with an Agilent 1200 HPLC system (Agilent Technologies, Santa Clara, CA, USA). It was equipped with a quaternary pump, an autosampler, a multi-wavelength UV detector operated at 280 and 330 nm, and a thermostatically controlled column compartment maintained at 35 °C. Chromatographic data were acquired and processed using HPLC 2D ChemStation/Chem32, Software B.04.03 (32 bits) G2170BA TL04041172, TL040426EF, (Agilent Technologies, Santa Clara, CA, USA), which enabled automatic recording of the retention times and integrated peak areas. Separation was carried out with a Zorbax Eclipse XDB-C18 analytical column (Agilent Technologies). Identification of phenolic compounds in *Ulva lactuca* and *Enteromorpha intestinalis* was based on comparison of the observed retention times with those of authentic reference standards analyzed at a concentration of 0.05 mg/mL. The reference standards and corresponding retention times were as follows: benzoic acid, 9.468 ± 0.098 min; caffeic acid, 8.281 ± 0.070 min; ellagic acid, 15.303 ± 0.030 min; ferulic acid, 8.865 ± 0.060 min; gallic acid, 0.990 ± 0.030 min; gentisic acid, 4.272 ± 0.009 min; *p*-hydroxybenzoic acid, 5.933 ± 0.006 min; *p*-coumaric acid, 15.971 ± 0.063 min; protocatechuic acid, 3.130 ± 0.008 min; salicylic acid, 15.952 ± 0.030 min; vanillic acid, 6.919 ± 0.050 min.

#### 4.7.3. Vitamin Content Determination

Vitamin content was determined in both aqueous and hydroalcoholic extracts obtained from the investigated algal species. Analytical procedures appropriate for each vitamin were applied in accordance with the AOAC guidelines [124]. Vitamin C, expressed as ascorbic acid, was determined in the aqueous algal extracts using the iodometric titration method described by Cadar (2023) [39]. The method is based on the oxidation of ascorbic acid to dehydroascorbic acid by iodine, accompanied by the reduction of iodine to iodide ions. The titration proceeds until all ascorbic acid present in the sample has been oxidized, after which excess iodine indicates the endpoint of the reaction. Retinol (vitamin A) and tocopherol (vitamin E) were determined by the HPLC technique. Analyses were performed using an Agilent 1100 Series HPLC system (Agilent Technologies, Santa Clara, CA, USA) equipped with a diode-array detector (DAD) and a Zorbax Eclipse XDB-C18 column. Vitamin A was detected at 325 nm and exhibited a retention time of approximately 4 min. Vitamin E was monitored at 293 nm, with a retention time of approximately 20 min [132]. The content of riboflavin (vitamin B2) and thiamine (vitamin B1) was evaluated by a reliable and reproducible UV–VIS spectrophotometric method, which allows for the simultaneous determination of both vitamins in the same sample. The analyses were performed using a Shimadzu UV-1601 spectrophotometer (Shimadzu Corporation, Kyoto, Japan), and the concentrations were calculated by the isobestic regression method. The spectrophotometric determinations were performed at wavelengths of 450 nm for thiamine and 410.5 nm for riboflavin [133]. Pyridoxine (vitamin B_6_) and niacin (vitamin B_3_) were measured by UV–VIS spectrophotometry using a Jasco V-530 spectrophotometer (JASCO Corporation, Tokyo, Japan). The method relies on the reaction of the vitamins with a potassium iodide–potassium iodate reagent in an aqueous medium, resulting in the formation of yellow triiodide ions. The intensity of the resulting absorption was proportional to the concentration of the analyte [134]. For vitamin B_6_, absorbance was measured at 290 and 335 nm, whereas vitamin B_3_ was determined at 288 and 350 nm, according to the method described by Khateeb (2015) [135].

### 4.8. Obtaining Biomaterials from Jellyfish Collagen and Seaweed

#### 4.8.1. Preparation of the B1 and B2 Collagen–Green Algae Biocomposites

The preparation of the new composite formulations was adapted from the method described by Pesterau (2025) for jellyfish collagen–brown algae biocomposites [35]. Two formulations containing type I marine collagen peptides and green algal extracts were developed: B1 and B2. In the first stage, the jellyfish collagen peptide extract (JPC) obtained from *Rhizostoma pulmo* was redissolved in 30 mL of double-distilled water. The pH of the resulting solution was adjusted to 9.0 by the gradual addition of 1 M NaOH or 1 M HCl, as required. In the second stage, the hydroalcoholic green algal extracts were incorporated into the collagen peptide solution at a concentration of 20%. The *Ulva lactuca* extract (ALGU) was used to prepare B1 (JPC–ALGU 20%), whereas the *Enteromorpha intestinalis* extract (ALGE) was used to prepare B2 (JPC–ALGE 20%). Each new formula was maintained under continuous magnetic stirring at 50 °C for 30 min to facilitate complete homogenization of the components and promote interactions between the collagen peptides and algal compounds. Following homogenization, the suspensions were centrifuged at 10,000 rpm for 15 min. The resulting hydrogel material was collected and subsequently divided into two portions for each formulation. The first fraction was lyophilized to obtain a dry material suitable for subsequent physicochemical and biological analyses. The second fraction was retained as a collagen peptide–algal extract hydrogel and stored under refrigerated conditions until use in the subsequent experimental procedures.

#### 4.8.2. Obtaining Films from Jellyfish Collagen Type I and Green Seaweed

Collagen films, also referred to as membranes, were prepared from *Rhizostoma pulmo* jellyfish collagen hydrogels incorporating hydroalcoholic extracts of green algae. The collagen hydrogel and algal extract were combined at a mass ratio of 4:1, after which 0.105 g of glycerol was added as a plasticizing agent. The mixtures were manually homogenized, and the pH was adjusted to 7.5. The resulting formulations were cast onto polyethylene sheets and dried at room temperature (24 °C) and 65% relative humidity for 72 h. The color of the films was determined by the incorporated algal extract and showed a degree of spatial variation. This non-uniform appearance was attributed to differences in layer thickness and to the intrinsic heterogeneity of the gels, which exhibited slight syneresis under mildly alkaline conditions at pH 7.5. In comparison, the gels were more homogeneous under acidic conditions at pH 3. Small air bubbles were also observed within the membranes. Their presence was associated with the high viscosity of the initial gels, which hindered the complete removal of entrapped air before casting and drying. After drying, the membranes were sterilized by exposure to ultraviolet radiation and stored in hermetically sealed polyethylene bags. These collagen-based membranes have potential applications in tissue regeneration [136].

#### 4.8.3. Preparation of Collagen Matrices from Type I Collagen Extracted from *Rhizostoma pulmo* Jellyfish and Green Seaweed

Porous collagen matrices were obtained by lyophilizing hydrogels prepared from type I collagen derived from *Rhizostoma pulmo* jellyfish and hydroalcoholic extracts of green algae. To obtain denser matrices with lower porosity and permeability, which may reduce the rate of in vivo resorption and provide a more gradual release of incorporated bioactive compounds, a significant collagen concentration is generally required [137]. For this purpose, the jellyfish collagen peptide component (JPC) was chemically cross-linked using glutaraldehyde. The efficiency of the cross-linking reaction may be influenced by several factors, including pH, solvent composition, reagent concentration, and reagent purity. The reaction is generally favored near neutral pH, where free, non-protonated amino groups are available to react with glutaraldehyde and form covalent cross-links within the collagen structure. Before cross-linking, the JPC preparation was conditioned for 12 h at 4 °C in the presence of an ammonia solution, and the pH was adjusted to 7.5. Glutaraldehyde was then added at a concentration corresponding to 0.1% of the collagen mass, and the reaction mixture was maintained for 24 h at 4 °C. The cross-linked JPC preparation was subsequently combined with each green algal extract at a collagen-to-algal extract mass ratio of 4:1. The resulting JPC–ALG hydrogel formulations were transferred into stainless-steel trays as layers approximately 5 mm thick. The thickness of the cast hydrogel layers determined the final thickness of the porous matrices. Lyophilization was performed using a Labconco freeze-dryer (Labconco Corporation, Kansas City, MO, USA). The process was conducted at a pressure of 0.18 mbar according to the following temperature program: the chamber temperature was decreased from 35 °C to −70 °C over 1 h; maintained at −70 °C for 5 h; increased to 5 °C over 10 h; maintained at 20 °C for 10 h; and subsequently maintained at 35 °C for 48 h. After completion of the 74 h lyophilization cycle, the trays were removed and the porous matrices were recovered. The collagen–algae matrices were sterilized by ultraviolet irradiation and stored in hermetically sealed polyethylene bags. Such porous collagen-based matrices are of considerable interest for tissue engineering and regenerative medicine applications [138].

### 4.9. Rheological Analysis of the New Biocomposites B1 and B2

The rheological properties of the B1 and B2 formulations were analyzed to assess their structural stability and flow behavior, which are important parameters for evaluating their suitability as semisolid pharmaceutical systems. Measurements were performed at a controlled temperature of 23 ± 0.1 °C. The equipment was a Haake VT 550 rheoviscosimeter (Thermo Fisher Scientific, Waltham, MA, USA) which has a sensor system suitable for medium-viscosity materials. Data processing was performed using RheoWin 4 software (Thermo Fisher Scientific, Waltham, MA, USA). The rheological study of hydrogels was conducted by recording rheograms and flow curves, which enabled evaluation of their flow behavior and identification of the rheological model best describing each formulation.

### 4.10. Biological Activities

#### 4.10.1. Antioxidant Activity

The DPPH test is a method based on the capture of the DPPH free radical (2 2-diphenyl-1-picrylhydrazyl), according to the protocol presented by Pesterau (2025), adapted from Brand’s procedures [35]. For the determinations, a 10 mM ethanolic DPPH solution was prepared, and a 5 mL aliquot was combined with 1 mL of each sample. The mixtures were incubated for 30 min. Subsequently, at 517 nm, the absorbance was read using an A. Krüss UV-Vis 6500 UV–VIS spectrophotometer (from A. Krüss Optronic GmbH, Hamburg, Germany). Antioxidant activity was calculated using the following calculation relationship:

(7) $$DPPH\left(\%\right)=\frac{A_{control}-A_{sample}}{A_{control}}\times 100$$

where A_sample corresponds to the absorbance of the samples to be analyzed, and A_control represents the absorbance of the control sample. Ascorbic acid (Merck KGaA, Darmstadt, Germany) was used as a reference standard (positive control), due to its well-known antioxidant properties. Based on the calibration curves, using nonlinear regression, IC_50_ values were also determined for all samples taken into work, these being reported as the mean ± SD of three replications.

Reducing power assay. The reducing capacity of the samples was evaluated using the reducing power assay, a method commonly applied to assess the electron-donating and antioxidant potential of phenolic and other reducing compounds. The method was based on the method Oyaizu (1986), reported by Pesterau (2025) [35]. For each determination, 1 mL of sample was mixed with 2 mL of 0.3 M phosphate buffer and 2 mL of 1% potassium ferricyanide solution. After incubation for 30 min at 55 °C, 2 mL of 10% trichloroacetic acid was introduced. Subsequently, a 1 mL aliquot was supplemented with 3 mL of distilled water and 1 mL of 0.1% ferric chloride solution. After 10 min of incubation, at 700 nm, the absorbance was read using an A. Krüss UV-Vis 6500 spectrophotometer (A. Krüss Optronic GmbH, Hamburg, Germany). Ascorbic acid solution supplied by Merck KGaA (Darmstadt, Germany) was used as the standard reference.

#### 4.10.2. Assessment of Antimicrobial Activity

The antimicrobial activity of all investigated materials, including the *Rhizostoma pulmo* collagen peptide extract, the green algal extracts, and the B1 and B2 biocomposites, was evaluated using the agar well diffusion method. The procedure was adapted from the protocol described by Li (2018) [139]. Antimicrobial activity was evaluated by determining the diameters of the inhibition zones formed around the wells.

The antimicrobial spectrum was evaluated against two Gram-positive bacterial strains, *Staphylococcus aureus* (ATCC 25923) and *Staphylococcus epidermidis* (ATCC 12228), and three Gram-negative strains, *Klebsiella pneumoniae* (ATCC 13883), *Proteus mirabilis* (ATCC 25933) and *Escherichia coli* (ATCC 25922). The bacterial strains and culture media were obtained from the Sanitary Veterinary and Food Safety Directorate of Constanta, Romania. Chloramphenicol purchased from Merck KGaA (Darmstadt, Germany) was used as the positive control, whereas untreated microbial cultures served as the negative control. The growth medium contained 20 g agar, 5 g NaCl, 5 g peptone, and 3 g meat extract per liter of distilled water and was sterilized before use. Microbial suspensions were uniformly inoculated onto the surface of the solidified agar medium to obtain confluent lawn cultures, according to procedures adapted from Li (2018), Jeyanthi (2012), and Tajbakhsh (2011) [139,140,141]. Wells were prepared in the inoculated agar, and the test samples were added under aseptic conditions. The diameters of the inhibition zones were measured and used to evaluate the antimicrobial activity of the samples. The MIC was also determined for each tested material.

### 4.11. Biological Evaluation of New Composites B1 and B2 for Wound-Healing Applications

#### 4.11.1. Cell Viability and Proliferation

The biocompatibility of the developed formulations was evaluated by assessing cell viability and proliferation using two representative cell lines: BALB/3T3 clone A31 fibroblasts, obtained from the American Type Culture Collection (ATCC, Manassas, VA, USA), and HaCaT human keratinocytes, supplied by Cell Systems GmbH (Eppelheim, Germany). These cellular models help in studying the effects of formulations on fibroblasts involved in connective tissue repair and on keratinocytes responsible for restoring the epidermal barrier. The cultures were prepared using Dulbecco’s Modified Eagle Medium (DMEM) supplemented with 10% FBS (fetal bovine serum), 2 mM L-glutamine, and 1% penicillin–streptomycin solution (Merck KGaA, Darmstadt, Germany). The cultures were incubated in a humidified atmosphere containing 5% CO_2_. Prior to passaging, the cell monolayers were rinsed with PBS. The cells were enzymatically detached using a trypsin–EDTA solution according to the protocol described by Migone (2022) [12]. BALB/3T3 fibroblasts were seeded at densities of 1 × 10^4^ or 2.5 × 10^4^ cells/well, whereas HaCaT keratinocytes were seeded at densities of 3 × 10^4^ or 2 × 10^4^ cells/well, depending on the 24 or 48 h incubation period. Following exposure to the investigated samples, fresh culture medium containing 10% WST-1 reagent was added after removal of the original medium reagent. The cells were returned to the incubator for an additional 4 h under the same culture conditions. The quantity of formazan produced through the reduction of WST-1 by metabolically active cells was measured using a BioTek 800 TS microplate reader (Agilent Technologies, Santa Clara, CA, USA). Absorbance was recorded at 465 nm using 655 nm as the reference wavelength. The concentration range of 0.015–2 mg/mL was selected based on previous studies using related jellyfish collagen–algal biomaterials on BALB/3T3 and HaCaT cell lines [35,74]. Cell viability and proliferation were calculated from the absorbance values obtained for the treated samples relative to those of the untreated control cells.

#### 4.11.2. Scratch Test on BALB/3T3 Cells and HaCat Cells

The ability of the developed formulations to promote cell migration and in vitro wound closure was evaluated using a scratch assay adapted from the methods described by Migone (2022) and Fabiano (2021) [12,142]. The experiments were performed at the Constanța County Emergency Hospital, Romania, using BALB/3T3 fibroblasts and HaCaT human keratinocytes. BALB/3T3 fibroblast cells were dispensed into 12-well plates at a density of 1.25 × 10^5^ cells/well in DMEM supplemented with 1% FBS (fetal bovine serum). The experiment was conducted in a humidified atmosphere containing 5% CO_2_ at 37 °C, and complete confluence was reached after approximately 24 h. HaCaT keratinocytes were cultured under the same conditions and at the same seeding density, although complete monolayer confluence was achieved after approximately 48 h.

After formation of a confluent monolayer, a linear scratch was created in each well using a sterile 200 μL pipette tip to simulate an in vitro wound. Detached cells and cellular debris were removed by gently washing the wells three times with phosphate-buffered saline (PBS). Subsequently, 2 mL of the analyzed samples was added to each well to achieve a final sample concentration of 0.125 mg/mL. Cells maintained in culture medium without any test sample served as the negative control. Wound closure was monitored by capturing images immediately after scratch formation (0 h) and after 4 h, 24 h, and 48 h of incubation. Images were acquired using a Nikon Eclipse Ts2R inverted microscope (Nikon Corporation, Tokyo, Japan) equipped with a 4× objective. Cell migration and wound closure were quantified by comparing the remaining cell-free area at each observation time with the initial scratch area recorded at 0 h.

The confluence rate was calculated with the equation:

(8) $$Confluency\;rate\;\left(\%\right)=\;\frac{Area\;T_{0}\;-\;Area\;T}{Area\;T_{0}}\;\times \;100$$

where Area T_0 represents the area at time 0, and Area T is for each sample at the end point. The confluence rate value was calculated to evaluate migration in both scratch tests performed [35].

### 4.12. Statistical Analysis

Statistical analyses were performed using IBM SPSS Statistics, version 16.0 (IBM Corporation, Armonk, NY, USA). Differences among the experimental groups, including the algal extracts, jellyfish collagen peptides, and developed biocomposite formulations, were evaluated using one-way analysis of variance (ANOVA). When the ANOVA indicated statistically significant differences, post hoc multiple comparisons were performed using Duncan’s multiple range test. Comparisons between two experimental groups were conducted using Student’s *t*-test. Statistical significance was established at *p* < 0.05 for all analyses. Unless otherwise stated, the experimental results were reported as the mean ± (SD) of three independent determinations. When a different number of replicates was used, the corresponding value of *n* was specified in the relevant methodology, table, figure, or Results Section. Data organization and graphical representations were prepared using Microsoft Excel 2019, included in Microsoft Office Professional Plus 2019 (Microsoft Corporation, Redmond, WA, USA).

## 5. Conclusions

The wound-healing process remains a problem that is not limited to repairing tissue integrity, reducing pain and costs and minimizing complications. Recently, the emergence of innovative treatments based on natural products, which generate optimized pharmaceutical formulations with low toxicity, improved efficacy and compliance, is a direction of interest for the medical field. In this context, we should reconsider harnessing resources that offer a wealth of bioactive compounds, such as the Black Sea. The use of collagen derived from invertebrate organisms and other natural compounds such as those extracted from seaweed represents a trend that can help address major medical challenges.

In this regard, the combination of collagen peptides from the jellyfish *Rhizostoma pulmo* with hydroalcoholic extracts from green seaweed, *Ulva lactuca* and *Enteromorpha intestinalis*, led to the obtaining of biocomposites B1 and B2, which demonstrated improved biological performances compared to the individual ingredients. Their antioxidant, antimicrobial and wound-closure activities support their potential use in wound-healing and tissue-engineering applications, in particular by facilitating cell migration and contributing to limiting oxidative damage and microbial contamination. However, the present results are based primarily on in vitro data obtained from fibroblast and keratocyte cell lines. Further in vivo studies are needed to confirm safety, tissue response, therapeutic efficacy, long-term stability, degradation behavior, dose-dependent cytotoxicity and interactions with damaged tissues. The resulting collagen–algae biomaterials may also be suitable for applications in skin and mucosal regeneration, tissue engineering and controlled drug delivery.

Future research should focus on optimizing extraction and processing methods, improving reproducibility, and establishing standardized quality-control procedures. Adequate regulatory frameworks will also be necessary to support the sustainable use of jellyfish and marine algae while protecting marine ecosystems. Overall, these marine-derived materials represent promising candidates for the development of alternative wound-healing and tissue-engineering products, provided that their safety and efficacy are confirmed through further preclinical and clinical studies.

## Figures and Tables

**Figure 1:**
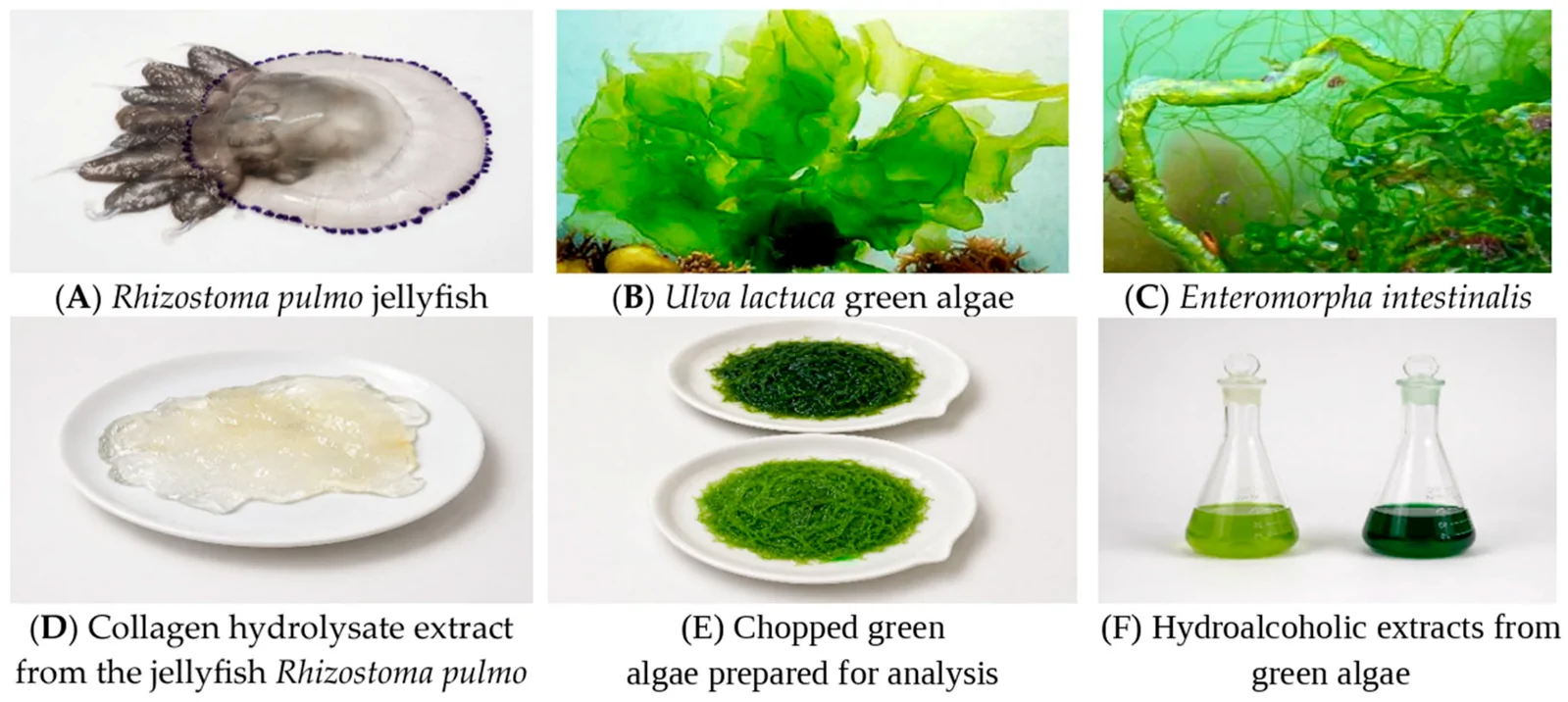
*Rhizostoma pulmo* jellyfish and green algae from the Black Sea (*Ulva lactuca* and *Enteromorpha intestinalis*), together with the extracts obtained from these marine resources: Figure 1B,C are from [39].

**Figure 2:**
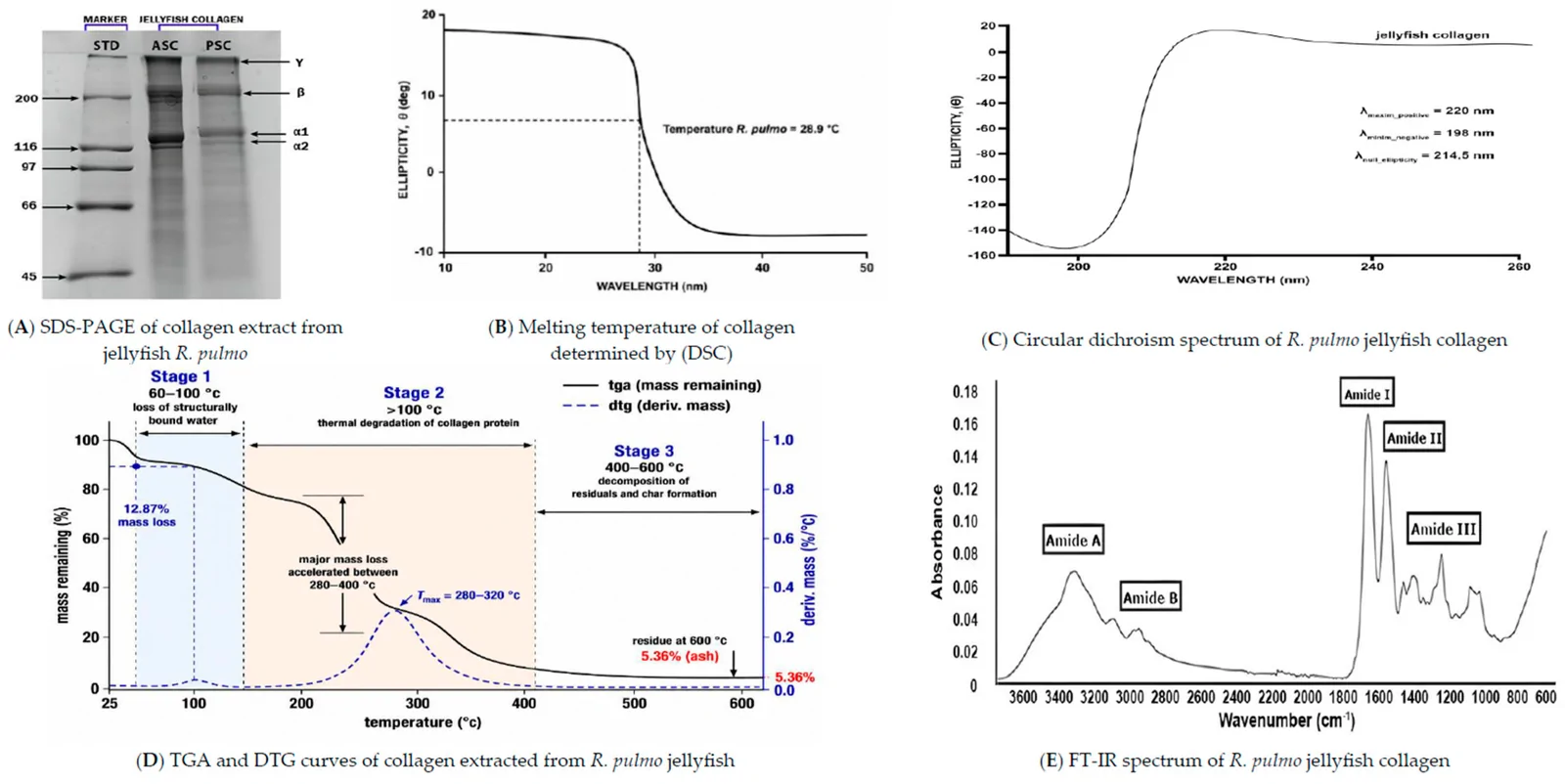
Physicochemical results from analysis of collagen: (**A**) SDS-PAGE analysis of *Rhizostoma pulmo* jellyfish; (**B**) Differential Scanning Calorimetry (DSC) of *Rhizostoma pulmo* jellyfish; (**C**) circular dichroism spectrum (CD spectrum) of *Rhizostoma pulmo* jellyfish; (**D**) thermogravimetric analysis of jellyfish collagen from *Rhizostoma pulmo*; (**E**) FT-IR spectrum for *Rhizostoma pulmo*.

**Figure 3:**
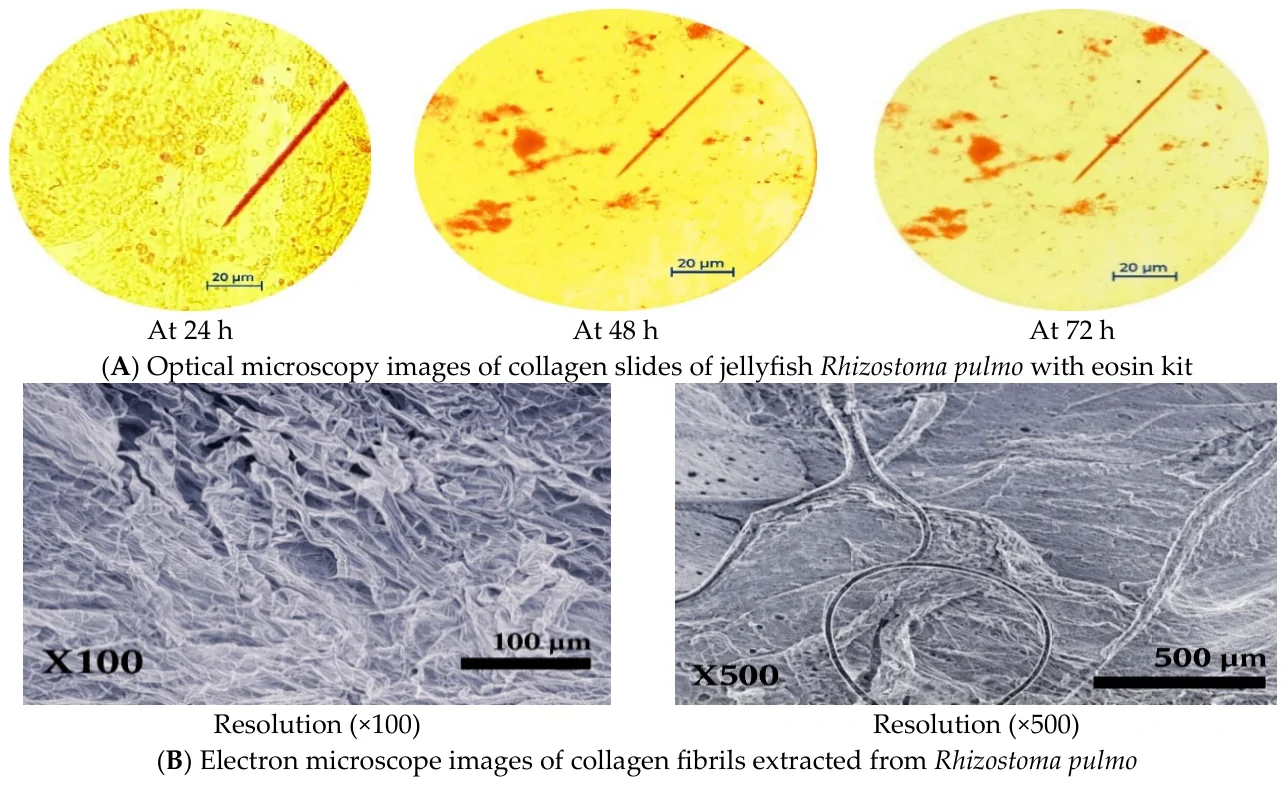
Optical and electron microscopy images for jellyfish collagen: (**A**) optical microscopy images recorded after 24, 48, and 72 h using eosin staining; (**B**) scanning electron microscopy (SEM) images showing the fibrillar morphology of the extracted collagen at ×100 and ×500 magnification.

**Figure 4:**
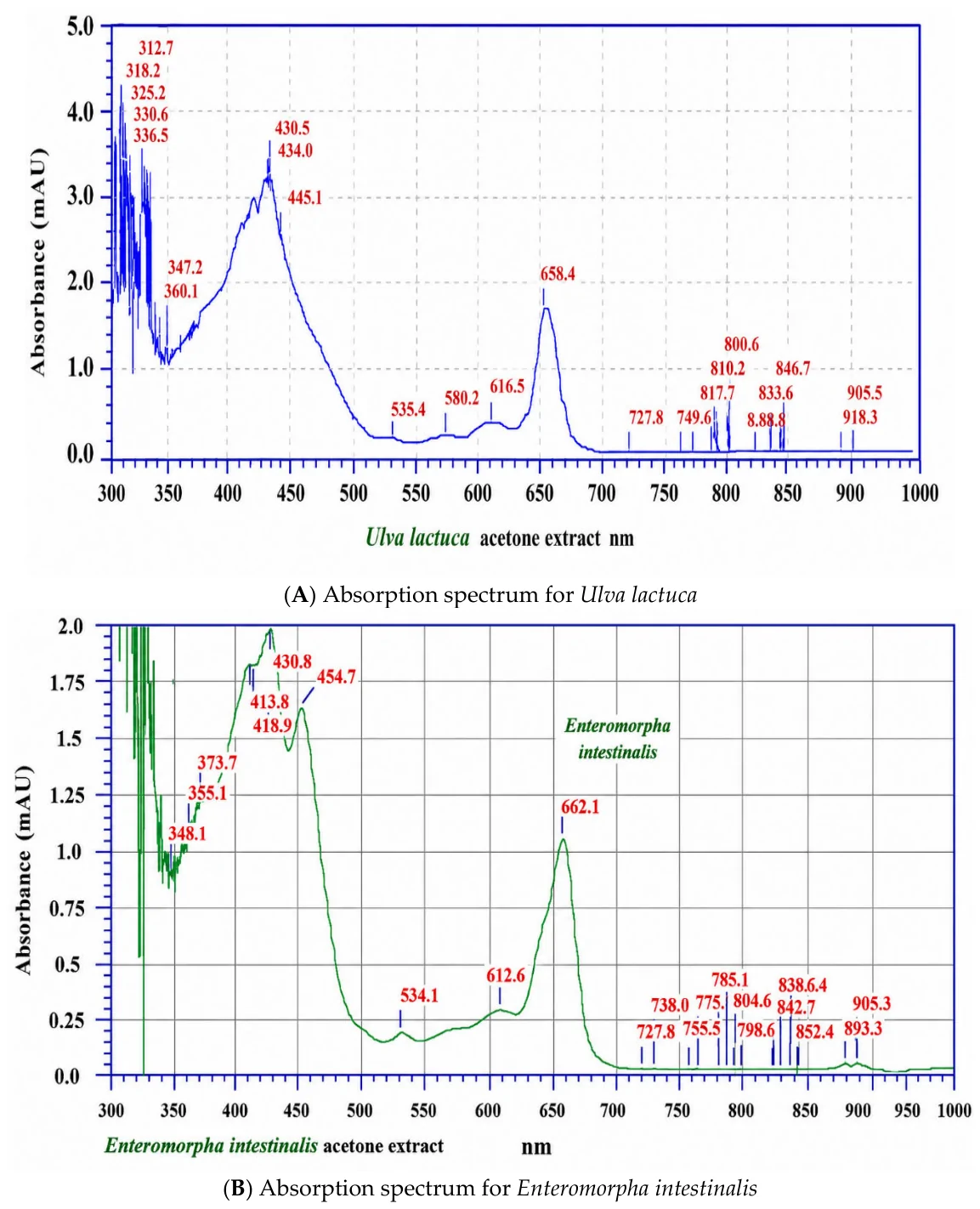
UV-VIS spectra for the two green algae: (**A**) absorption spectrum for *Ulva lactuca*; (**B**) absorption spectrum for *Enteromorpha intestinalis*.

**Figure 5:**
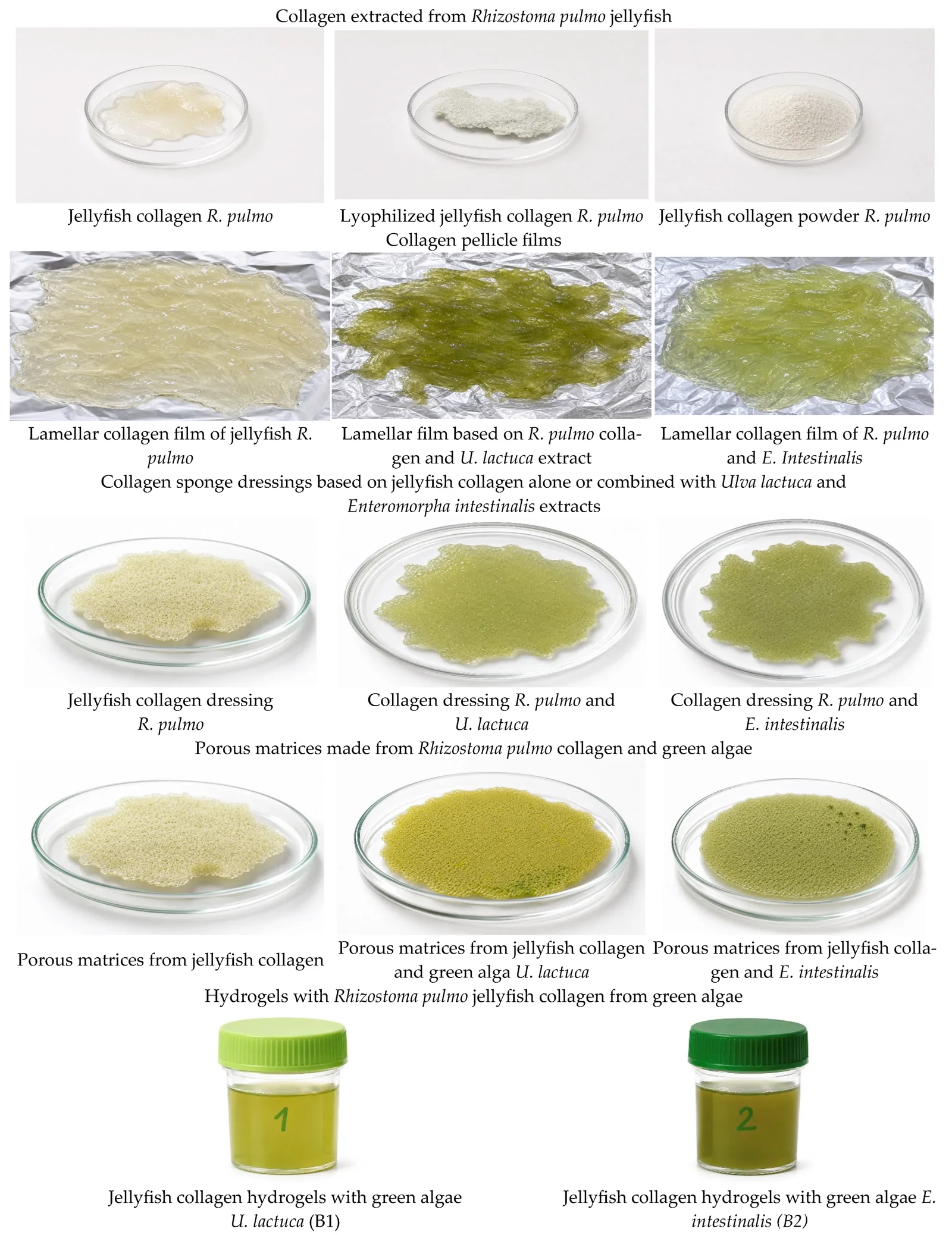
Biomaterials from jellyfish collagen from *Rhizostoma pulmo* with marine green algae ALGU and ALGE.

**Figure 6:**
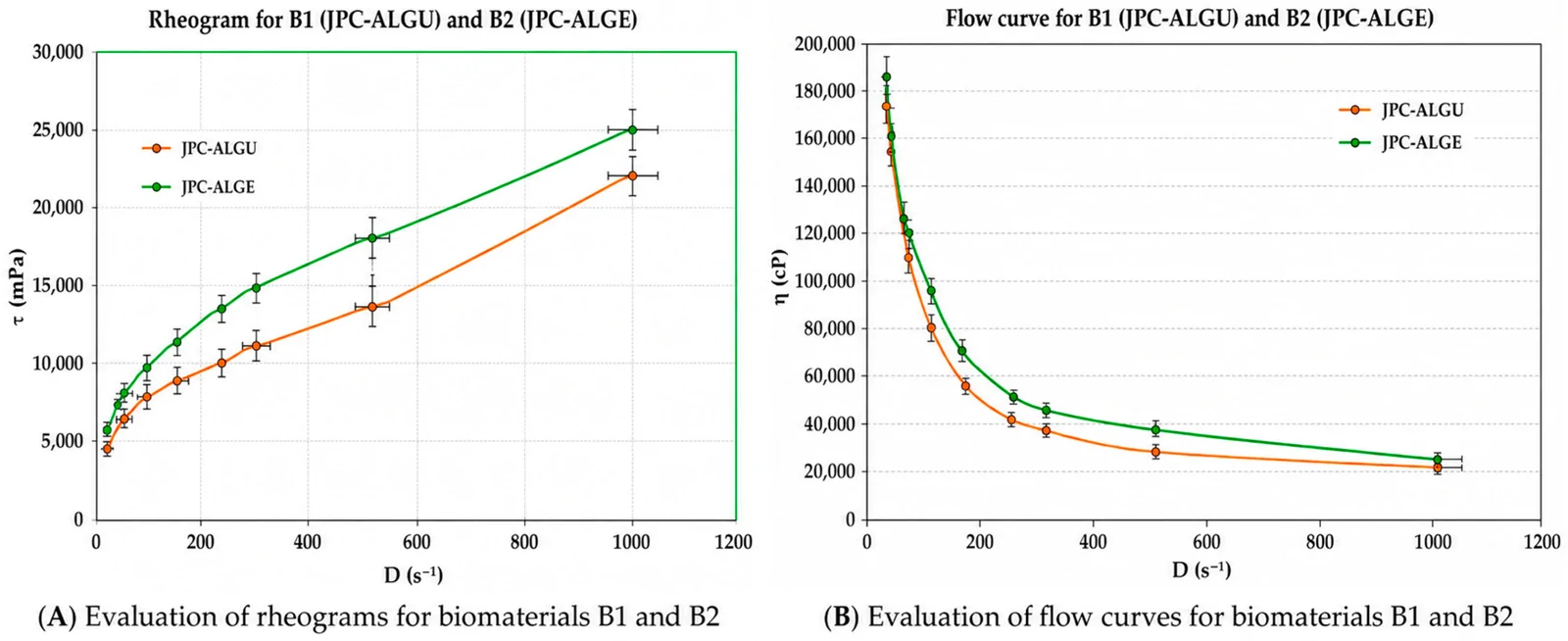
Rheological evaluation of two new materials B1 and B2.

**Figure 7:**
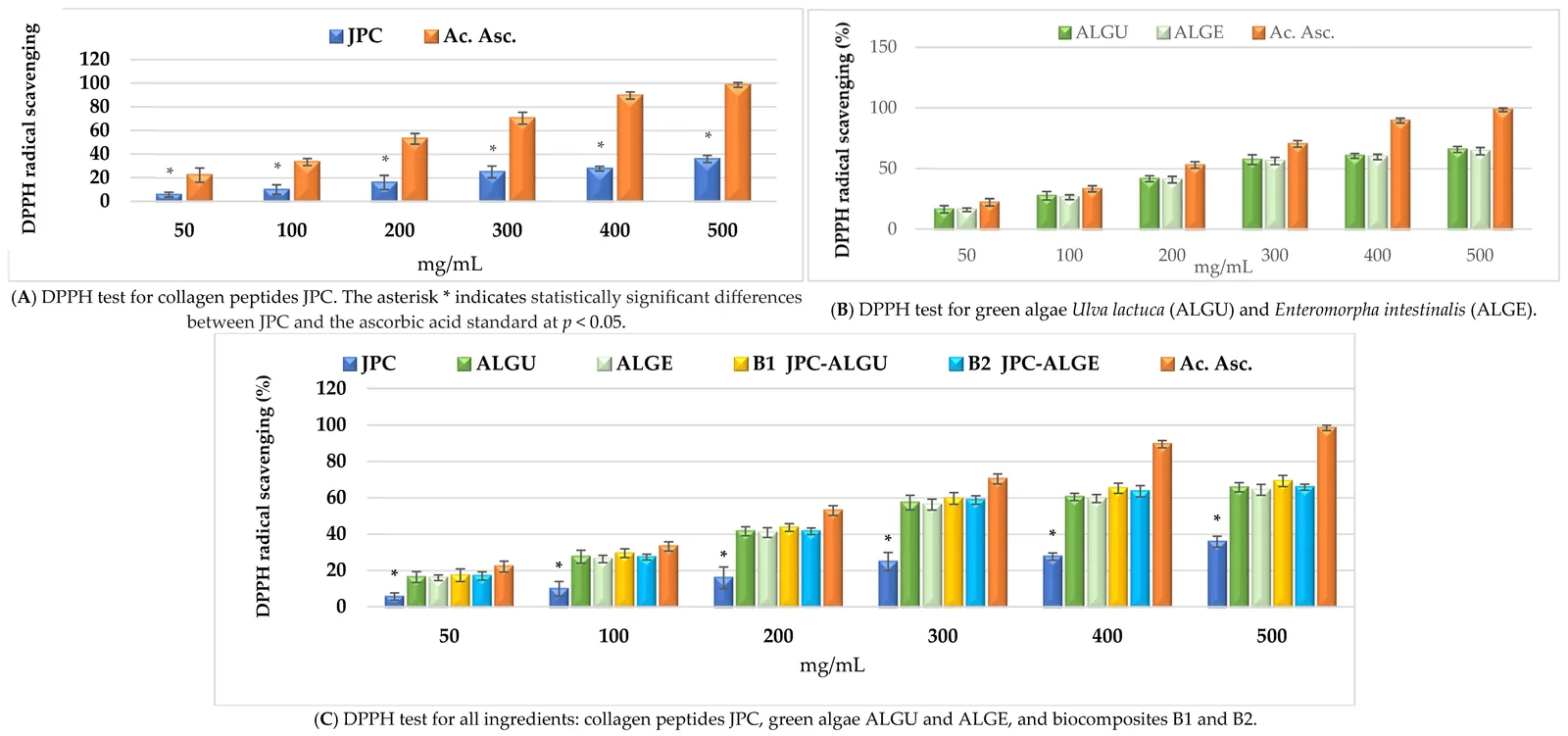
Results for antioxidant activity in DPPH assay for collagen hydrogels prepared from *Rhizostoma pulmo* JPC and green algae *Ulva lactuca* and *Enteromorpha intestinalis* from the Black Sea (*n* = 3; the asterisk * indicates statistically significant differences between JPC and the ascorbic acid standard at * *p* < 0.05.

**Figure 8:**
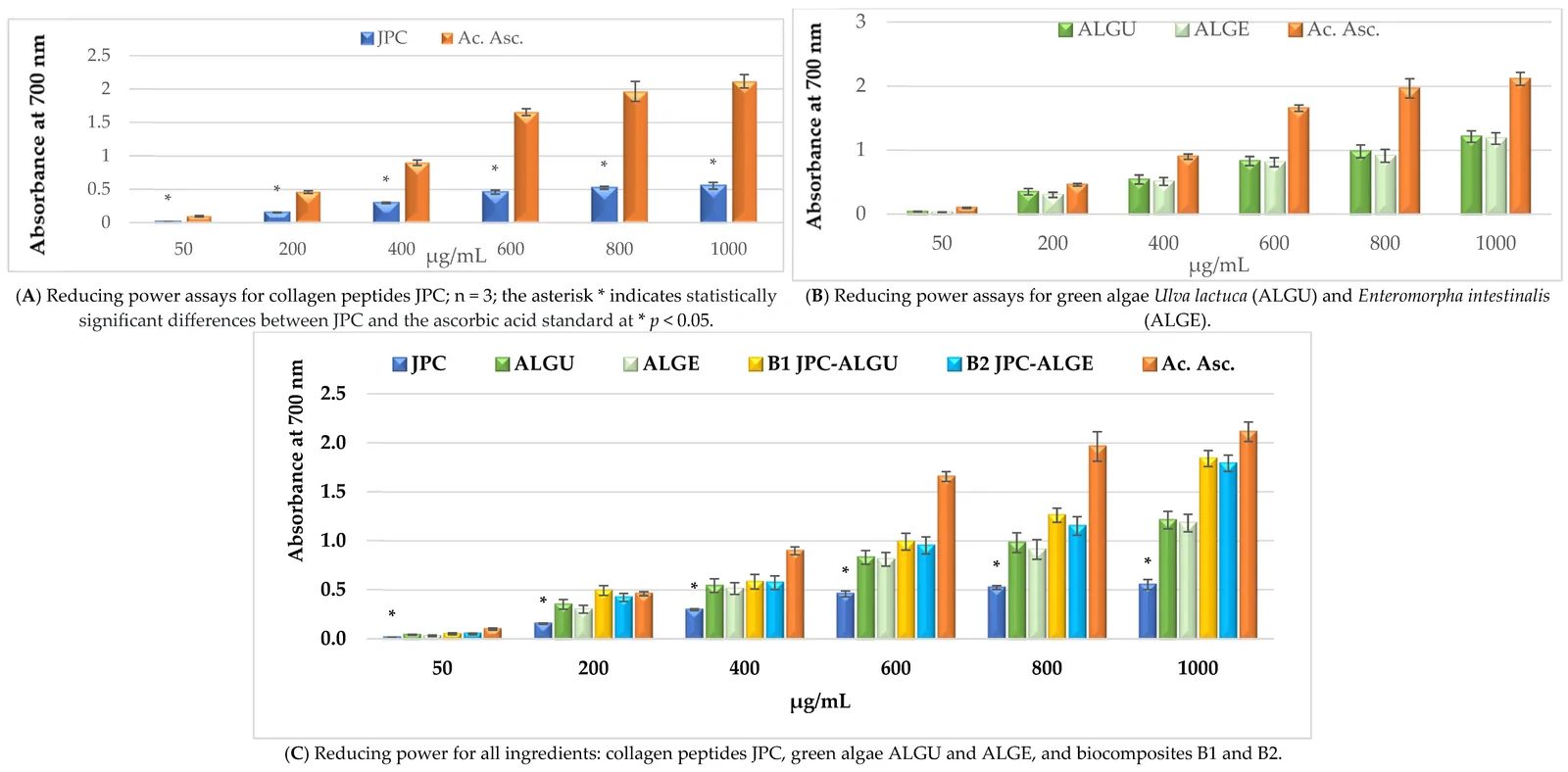
Antioxidant activity determined using the reducing power assay for collagen hydrogels prepared from *Rhizostoma pulmo* jellyfish collagen peptides (JPC) and extracts of the Black Sea green algae *Ulva lactuca* and *Enteromorpha intestinalis*; *n* = 3; the asterisk * indicates statistically significant differences between JPC and the ascorbic acid standard at * *p* < 0.05.

**Figure 9:**
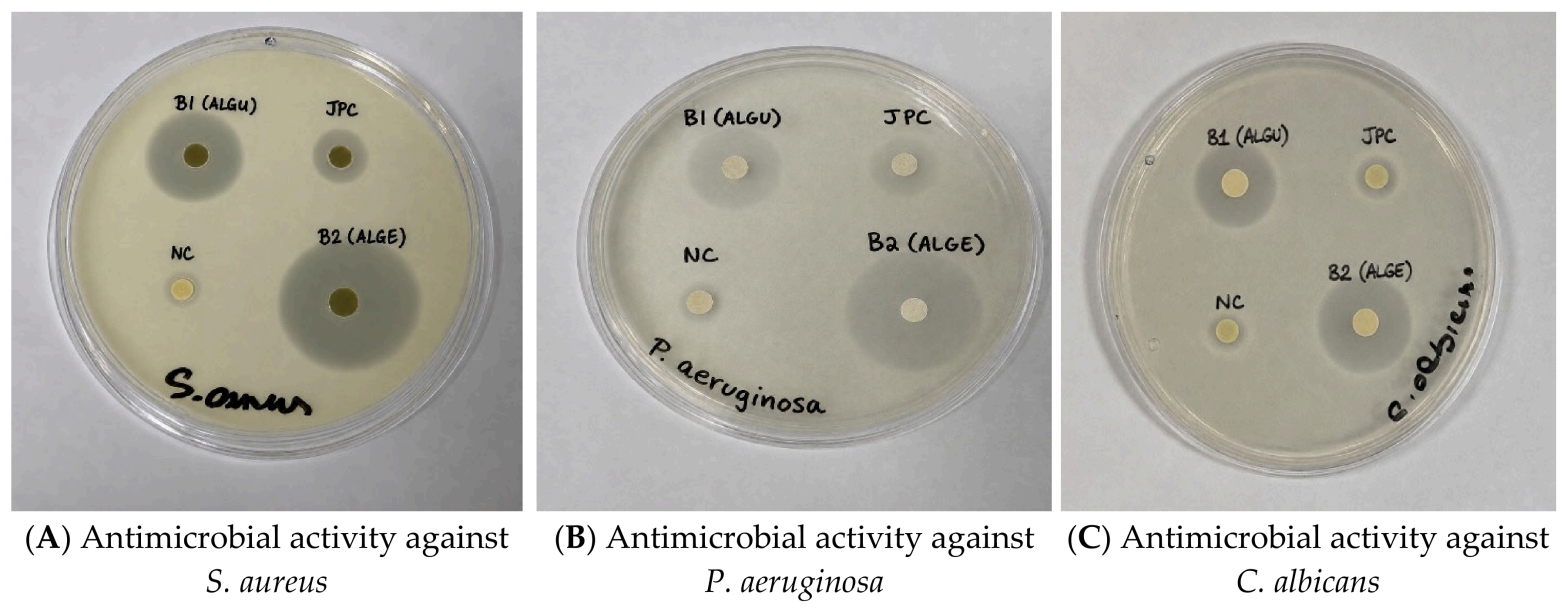
Antimicrobial activity of JPC extract from *R. pulmo*, *B1 *and* B2* against *S. aureus*, *P. aeruginosa* and *C. albicans*; NC—negative control.

**Figure 10:**
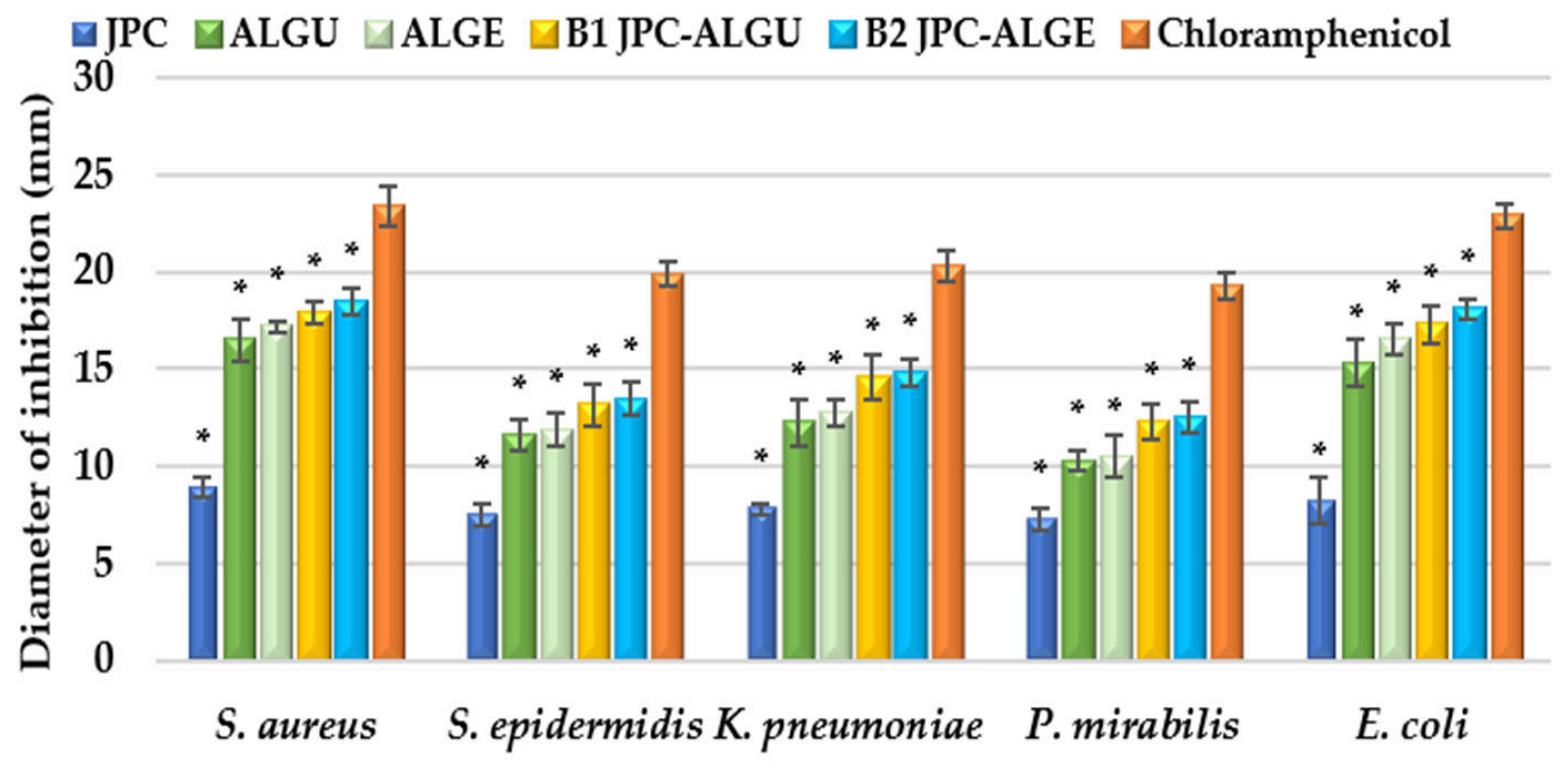
Antimicrobial activity of jellyfish collagen peptides (JPC), *Ulva lactuca* extract (ALGU), *Enteromorpha intestinalis* extract (ALGE), B1 and B2, expressed as inhibition-zone diameters. Data are presented as mean ± SD (*n* = 3). * *p* < 0.05 indicates statistically significant differences relative to the corresponding positive control.

**Figure 11:**
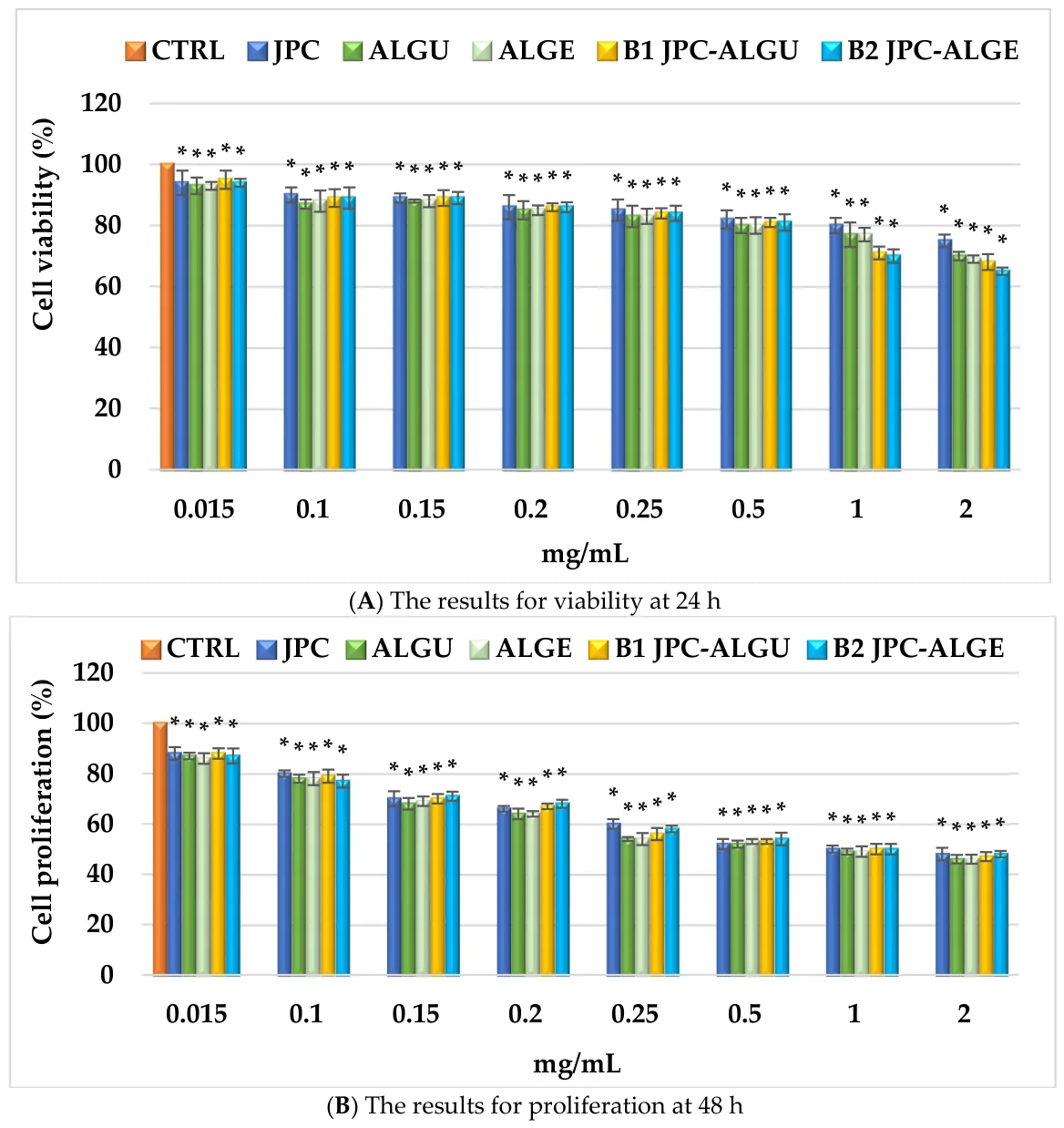
Variation in cytotoxicity on BALB/3T3 fibroblast cell line clone A31 treated with jellyfish collagen and the two biocomposites B1 and B2 over the concentration range of 0.015–2 mg/mL. (**A**) Results of the cell viability assay at 24 h. (**B**) Results of the proliferation assay at 48 h. The cytotoxicity test results are expressed as the mean ± standard deviation for 8 replicates. Statistically significant differences observed between all samples compared to the control sample are marked with an asterisk (*) at *p* < 0.05. The control sample consisted of untreated cells.

**Figure 12:**
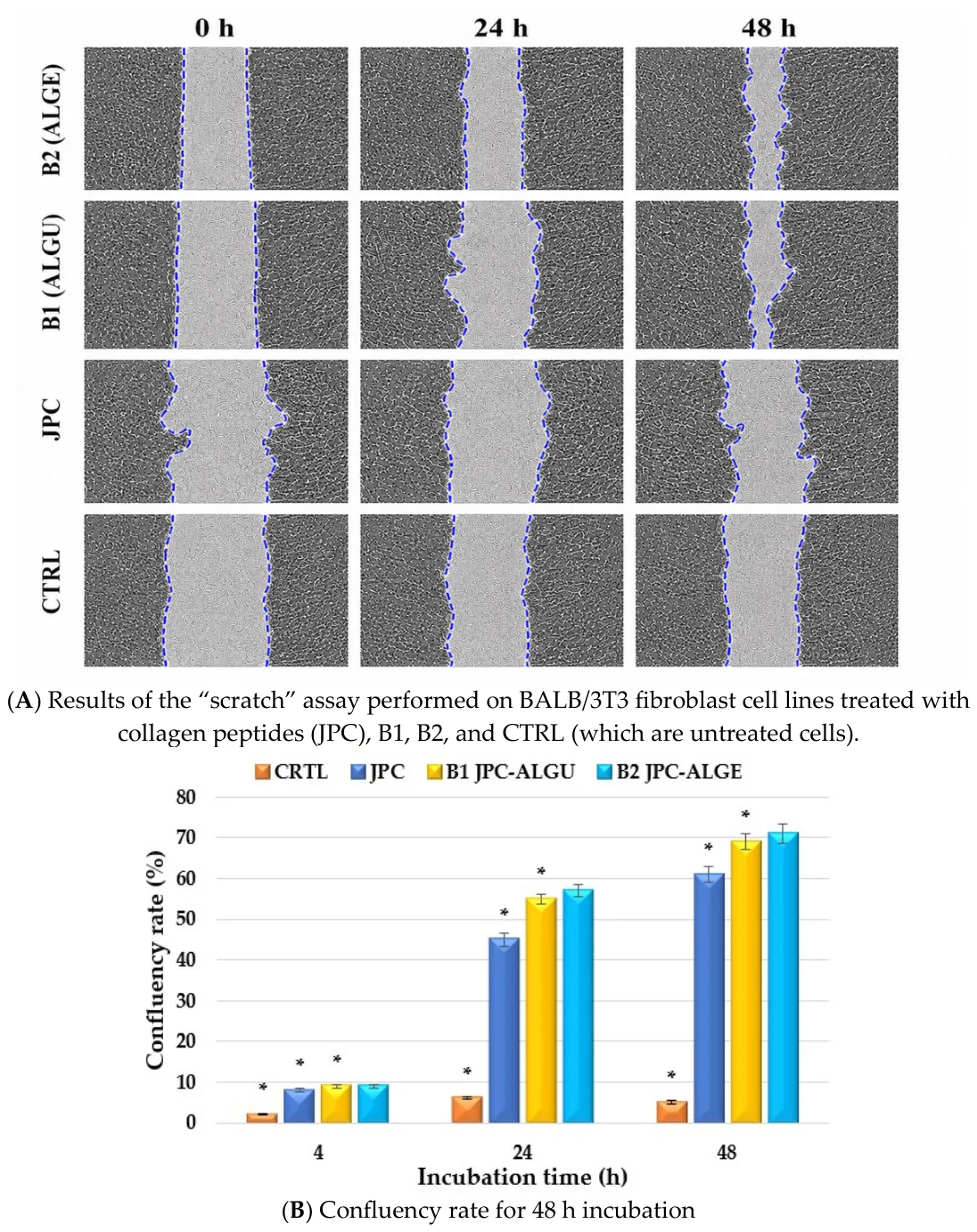
Scratch assay performed on BALB/3T3 fibroblasts from clone A31 treated with jellyfish collagen peptides (JPC), B1, B2 and CTRL. (**A**) Results of the scratch assay for fibroblasts, shown as representative micrographs (4× magnification); (**B**) confluence rate obtained after 48 h. Untreated cells were used as a control. Results are presented as the mean ± standard deviation (SD) of eight independent measurements (*n* = 8). Statistical significance relative to the untreated control is indicated by an asterisk (*) at *p* < 0.05.

**Figure 13:**
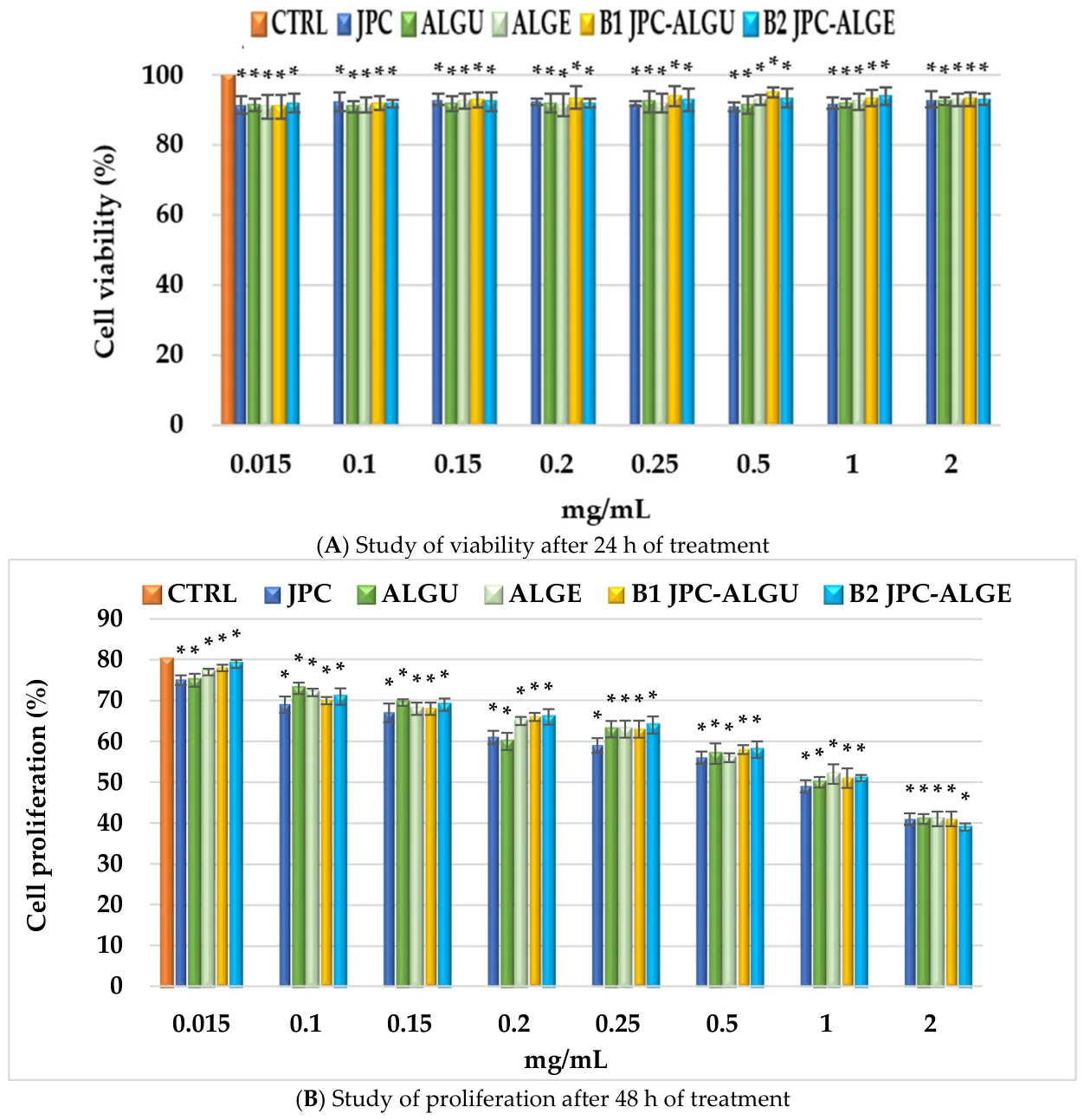
Variation in cytotoxicity tested on HaCaT human keratinocyte cell lines treated with jellyfish collagen peptides (JPC) and B1 and B2 biocomposites. (**A**) Viability assay after 24 h of treatment; (**B**) proliferation assay after 48 h of treatment. CTRL refers to untreated cells and was used as a control. Results are presented as mean ± standard deviation for *n* = 8. An asterisk (*) indicates statistically significant differences among all samples compared to the control at *p* < 0.05.

**Figure 14:**
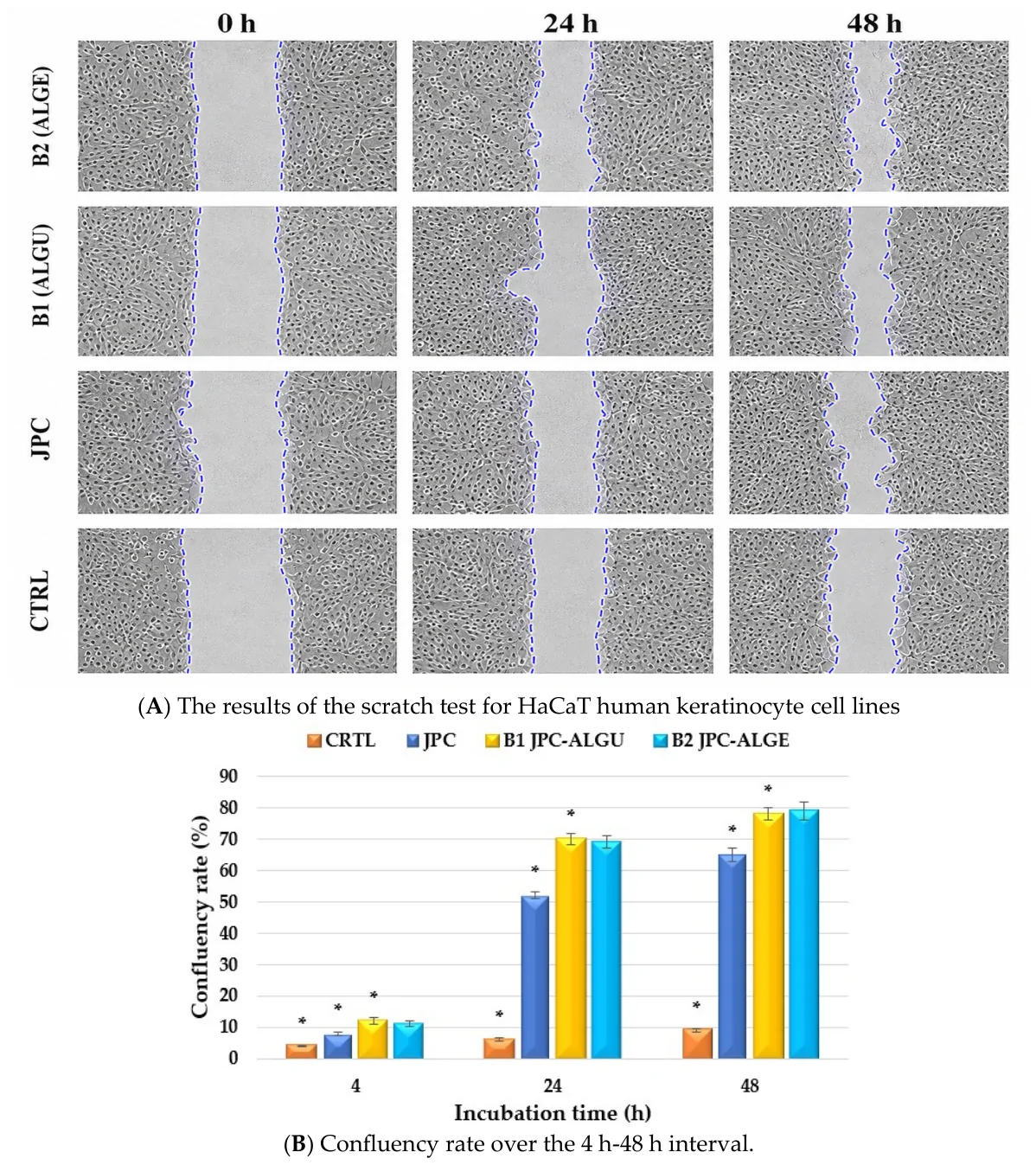
Scratch test performed on human HaCaT keratinocytes treated with jellyfish collagen peptides (JPC), B1, and B2. (**A**) The results of the scratch test for HaCaT human keratinocyte cell lines, presented as representative micrographs; (**B**) confluence rate over the 4–48 h time interval. CTRL represents the control group, which consists of untreated cells. The results represent the mean ± standard deviation for *n* = 8. Using one-way ANOVA and paired *t*-tests, statistically significant differences were indicated by an asterisk (*) for all samples compared to CTRL at *p* < 0.05.

**Table 1:** Proximate composition of jellyfish *Rhizostoma pulmo* from the Black Sea and of green algae *U. lactuca* and *E. intestinalis*.

Characteristics	Hydrogel Extract from JPC		Hydroalcoholic Green Algal Extracts			
	PSC (with 10% Pepsin)		*U. lactuca*		*E. intestinalis*	
		References [35]		References [39,40]		References [39,40]
Moisture (% D.W.)	18.6 ± 0.18	15.10 ± 0.1	10.23 ± 0.21	12.86 ± 1.01	11.65 ± 0.50	9.14 ± 2.93
Ash (% D.W.)	0.85 ± 0.15	0.55 ± 0.1	23.54 ± 0.54	18.38 ± 3.08	25.32 ± 0.68	30.28 ± 9.01
Proteins (% D.W.)	24.59 ± 1.89 B 31 ± 1.89 OA 61.25 ± 1.25 W	23.59 ± 2.89 B 32.21 ± 1.19 OA 60.48 ± 1.72 W	14.08 ± 1.92	12.18 ± 3.08	13.59 ± 0.83	12.10 ± 0.89
Collagen content (% D.W.)	59.5 ± 0.4 W	57.10 ± 0.6 W	*-*	-	-	-
Lipids (% DW)	4.6 ± 0.66 W	4.92 ± 0.81 W	2.56 ± 0.98	0.69 ± 0.06	1.68 ± 0.75	1.69 ± 0.47
Carbohydrates (% D.W.)	0.65 ± 1.28 W 0.28 ± 0.1 G	0.59 ± 1.25 W 0.25 ± 0.65 G	60.95 ± 2.02	54.95 ± 1.43	61.56± 1.98	52.36 ± 3.26
Total dietary fiber (% D.W.)	-	-	60.23 ± 1.20	59.50 ± 2.15	59.41 ± 1.97	52.36 ± 3.26
Insoluble fiber (% D.W.)	-	-	32.14 ± 1.23	33.59 ± 3.25	31.09 ± 0.92	31.54 ± 1.58
Soluble fiber (% D.W.)	-	-	29.56 ± 1.66	25.91 ± 1.25	27.31 ± 1.5	20.82 ± 0.56
Total phenolic content TPC (mg GAE/100 g D.W.)	1.54 ± 0.29	2.07 ± 0.31 [20]	418.50 ± 0.93	417.6 ± 1.56	415.62 ± 1.34	413.50 ± 1.26
Total flavonoid content TFC (mg QCE/100 g D.W.)	-	-	18.6 ± 1.15	16.60 ± 1.65	15.1 ± 1.25	14.10 ± 1.68

Percentages of dry weight—(% D.W.); whole—W; bell—B; oral arms—OA; gonad—G.

**Table 2:** Composition of amino acids from *Rhizostoma pulmo* jellyfish collagen.

Amino Acids	*Rhizostoma pulmo* Jellyfish		
	Our Values	References	
	Residues/1000 Residues		%
Essential amino acids (EAAs)			
		Reference [32]	Reference [35]
Glycine (Gly)	30.7	29.34	33.40
Glutamic acid (Glu)	14.8	13.46	15.20
Leucine (Leu)	8.1	6.35	8.60
Threonine (Thr)	6.5	3.18	5.25
Lysine (Lys)	6.4	4.62	6.30
Arginine (Arg)	5.8	5.63	6.20
Valine (Val)	3.5	2.80	4.91
Proline (Pro)	3.2	2.97	3.90
Hydroxyproline (Hyp)	3.1	4.82	3.65
Tyrosine (Tyr)	3.2	1.77	3.90
Tryptophan (Trp)	2.9	4.72	2.80
Cystine (Cys)	1.8	-	1.20
Histidine (His)	0.4	-	0.60
Non-essential amino acids (NEAAs)			
Alanine (Ala)	8.2	10.38	6.90
Aspartic acid (Asp)	9.5	10.91	6.65
Serine (Ser)	0.9	-	1.70

In both studies, the analyses were performed using whole-body tissue.

**Table 3:** Results obtained for the content of individual polyphenolic compounds.

Phenolic Compound (mg/100 g f.w.)	*Ulva lactuca*		*Enteromorpha intestinalis*	
	Mean Values ± SD	Reference [41]	Mean Values ± SD	Reference [41]
Caffeic acid	12.98 ± 0.15	13.1 ± 0.12	16.30 ± 0.13	15.70 ± 0.11
Vannilic acid	2.42 ± 0.04	1.6 ± 0.20	1.90 ± 0.01	1.80 ± 0.25
Protocatechuic acid	72.55 ± 0.30	70.8 ± 0.21	64.80 ± 0.10	55.60 ± 0.11
p-Hydroxybenzoic acid	3.8 ± 0.20	2.3 ± 0.11	4.20 ± 0.02	5.50 ± 0.31
Benzoic acid	1.8 ± 0.04	1.5 ± 0.11	1.25 ± 0.30	0.91 ± 0.05
Ellagic acid	3.89 ± 0.38	1.1 ± 0.61	2.90 ± 1.59	1.50 ± 0.40
Gentisic acid	128.96 ± 0.20	122.5 ± 0.61	98.20 ± 0.16	87.51 ± 0.80
Salicylic acid	3.6 ± 0.55	1.35 ± 0.03	1.80 ± 0.66	1.20 ± 0.12
Ferulic acid	3.20 ± 0.03	1.15 ± 0.05	2.87 ± 0.17	1.05 ± 0.12
p-Coumaric acid	2.33 ± 0.03	1.93 ± 0.03	2.12 ± 0.23	1.33 ± 0.03
Gallic acid	95.29 ± 0.20	90.51 ± 1.51	88.70 ± 0.20	78.25 ± 0.20

**Table 4:** The values obtained from the analysis of vitamin content.

Vitamin Type (mg/100 g Dry Powder)	*Ulva lactuca*		*Enteromorpha intestinalis*	
	Mean Value ± SD	Reference [39]	Mean Value ± SD	Reference [39]
Vitamin B1 (Thiamine)	3.981 ± 0.14	3.72 ± 0.25	3.80 ± 0.85	3.95 ± 0.52
Vitamin B2 (Riboflavin)	1.265 ± 0.08	0.99 ± 0.07	1.047 ± 0.04	0.97 ± 0.02
Vitamin B3 (Niacin)	3.558 ± 0.47	2.97 ± 0.28	2.194 ± 0.35	1.84 ± 0.45
Vitamin B6 (Pyridoxine)	0.846 ± 0.12	0.58 ± 0.12	0.687 ± 0.47	0.53 ± 0.66
Vitamin A (Retinol)	0.748 ± 0.02	0.57 ± 0.06	0.627 ± 0.05	0.49 ± 0.05
Vitamin E (Tocopherol)	9.956 ± 0.15	8.13 ± 1.03	10.264 ± 0.97	9.93 ± 0.83
Vitamin C (Ascorbic acid)	145.69 ± 1.05	143.63 ± 0.9	140.080 ± 0.90	136.16 ± 0.8

**Table 5:** Biomaterials obtained from jellyfish collagen from the Black Sea.

Biomaterials	Color	Type of Physical Form
Collagen peptides from Rhizostoma pulmo jellyfish	White	powder
Membrane or Lamellar film from jellyfish collagen	White	film
Sponge from jellyfish collagen	White	showing porosity
Hydrogel B1 (JPC-ALGU 20%) obtained from collagen peptide jellyfish with extract of *Ulva lactuca*	Light green–white	viscous gelatin
Hydrogel B2 (JPC-ALGE 20%) obtained from collagen peptide jellyfish with extract of *Enteromorpha intestinalis*	Light green–white	viscous gelatin

**Table 6:** MIC values of B2 against the investigated bacterial strains.

Type of Bacteria	MIC (µg/mL)		
	Jellyfish Collagen	B2 (JPC-ALGE 20%)	ALGE
*S. aureus*	25	25	25
*P. aeruginosa*	50	25	25
*C. albicans*	75	50	50
*E. coli*	50	25	25
*S. epidermidis*	50	50	50
*K. pneumoniae*	75	75	75
*P. mirabilis*	>100	>100	>100

## Data Availability

The original contributions presented in the study are included in the article; further inquiries can be directed to the corresponding authors.

## Abbreviations

The following abbreviations are used in this manuscript:

JPC	Jellyfish collagen peptides
JPC-ALGU	Composite jellyfish peptide–green algae *Ulva lactuca*
JPC-ALGE	Composite jellyfish peptide–green algae *Enteromorpha intestinalis*
DC	Dichroism circular spectrum
DSC	Differential scanning calorimetry
SDS-PAGE	Sodium dodecyl sulfate–polyacrylamide gel electrophoresis
TGA	Thermogravimetric analysis
FT-IR	Fourier-transform infrared spectroscopy
ASC	Acid-soluble collagen
PSC	Pepsin-soluble collagen
TPC	Total phenolic content
TFC	Total flavonoid content
SEM	Scanning electron microscopy
DPPH	2,2-diphenyl-1-picrylhydrazyl
MIC	Minimal inhibitory concentration
BALB/3T3	Fibroblast cell line
HaCaT	Keratinocyte cell line
ROS	Reactive oxygen species
EMC	Extracellular matrix
TGF-α	Transforming growth factor-alpha
TGF-β	Transforming growth factor-beta
FGF	Fibroblast growth factor
PDGF	Platelet-derived growth factor
VEGF	Vascular endothelial growth factor
